# Transcriptome Sequencing Reveals Potential Roles of *ICOS* in Primary Sjögren’s Syndrome

**DOI:** 10.3389/fcell.2020.592490

**Published:** 2020-12-04

**Authors:** Jing Luo, Xin Liao, Lihe Zhang, Xin Xu, Senhong Ying, Mengjiao Yu, Lixia Zhu, Suxian Lin, Xiaobing Wang

**Affiliations:** ^1^Rheumatology Department, The First Affiliated Hospital of Wenzhou Medical University, Wenzhou, China; ^2^Institute of Genomic Medicine, Wenzhou Medical University, Wenzhou, China; ^3^Rheumatology Department, Wenzhou Central Hospital, The Dingli Clinical Institute of Wenzhou Medical University, Wenzhou, China; ^4^Rheumatology Department, Wenzhou People’s Hospital, Wenzhou, China

**Keywords:** primary Sjögren’s syndrome, single gene GSEA, WGCNA, RT-qPCRl, *ICOS*, ELISA

## Abstract

Primary Sjögren’s syndrome (pSS) is a chronic systemic autoimmune disease characterized by exocrine gland damage and extraglandular involvements. To identify potential biomarkers for the early detection of pSS and to further investigate the potential roles of the biomarkers in the progression of pSS, our previous RNA sequencing data and four microarray data of salivary glands (SGs) were combined for integrative transcriptome analysis between pSS and non-pSS. Differential gene expression analysis, gene co-expression network analysis, and pathway analysis were conducted to detect hub genes, which were subsequently investigated in peripheral blood mononuclear cell (PBMC) and plasma. Correlation analysis, single-gene Gene Set Enrichment Analysis, and receiver operating characteristic (ROC) curve were applied to investigate the potential function of the hub genes and their classification capacity for pSS. A total of 51 common up-regulated genes were identified among different pSS cohorts. A key module was found to be the most closely linked to pSS, which was significantly associated with inflammation-related pathways. Seven overlapped hub genes (*ICOS*, *SELL*, *CR2*, *BANK1*, *MS4A1*, *ZC3H12D*, and *CCR7*) were identified, among which *ICOS* was demonstrated to be involved in most crucial immune pathways. *ICOS* was up-regulated not only in SGs but also in PBMC and plasma in pSS, and the expression of *ICOS* was closely associated with lymphocytic infiltration in SGs and disease activity of pSS patients. It showed a strong classification capacity with classic clinical index in SGs (ROC curve 0.9821) and significant distinct discrimination in PBMC (ROC curve 0.9107). These findings are expected to gain a further insight into the pathogenesis of pSS and provide a promising candidate for the early detection of pSS.

## Introduction

Primary Sjögren’s syndrome (pSS) is a chronic systemic autoimmune disorder characterized by exocrine gland damage caused by focal lymphocytic infiltration especially presenting as xerostomia and xerophthalmia in nearly 90% patients (Seror et al., 2010; Manuel et al., 2017). The disease affects females more frequently than males, with a ratio of incidence close to 10:1, particularly middle-aged women between 30 and 50 years (Ramos-Casals et al., 2015; Brito-Zeron et al., 2016). Besides the dysfunction of exocrine glands, extraglandular involvements are widely varied, ranging from mild non-specific symptoms including arthralgia, fatigue, and rash to severe systemic involvement, such as interstitial pneumonia, tubulointerstitial nephritis, neuropathy, and lymphoma, leading to poor prognosis and increased social burden (Ramos-Casals et al., 2012). Hence, there is an unmet need for in-depth investigation of the pathogenesis and a novel improved diagnostic marker for early intervention of the process of pSS.

The identification of biomarkers of pSS by high-throughput technologies may contribute to elucidating the molecular mechanism, improving on current classification criteria, and providing insight into any possible therapeutic targets (Aqrawi et al., 2017; Baldini et al., 2018). Zhang et al. (2019) found a series of distinct gene expression signatures in pSS and identified 19 hub genes, with some of them correlated with inflammatory response or interferon pathways. The RNA-seq analysis of salivary glands (SGs) also revealed that the expressions of *CCR7* and *CCL21* were markedly increased, which may assist in the recruitment of diverse immune cells to the SGs (Tandon et al., 2017). Moreover, *in vitro* experiments demonstrated that interleukin 17 (IL-17)-producing helper T cells (Th17)-like [CD4(+) CXCR5(+) CCR6(+)] T cells in circulation were found to be significantly higher in pSS than that in controls, and activated TH17-like cells could regulate follicular helper T cell (Tfh cell) differentiation and facilitate naïve B cells producing immunoglobulin (Li et al., 2012).

Weighted gene co-expression network analysis (WGCNA) is a method frequently used to explore functional pathways and candidate biomarkers through integrating gene expression and clinical data effectively (Langfelder and Horvath, 2008; Presson et al., 2008). In addition, WGCNA has been applied to construct gene co-expression network modules and identify hub genes in several autoimmune diseases, including rheumatoid arthritis (RA) (Ma et al., 2017), inflammatory bowel disease (IBD) (Li et al., 2016), and autoimmune thyroid disease (ATD) (Shao et al., 2018). Moreover, the gene expression profiles of more than 200 PBMC samples of pSS have been investigated, and WGCNA analysis was applied to explore the gene–network signature and potential functions of hub genes based on Gene Expression Omnibus (GEO) public database (Yao et al., 2019). Recently, Inamo et al. (2020) also identified *LINC00487* and *SOX4* as key genes associated with the dysregulation of B cells in pSS patients using WGCNA algorithm. These studies provided a convincing possibility that WGCNA can be effectively applied to identify hub genes as biomarkers for the early detection of pSS and facilitate a deep understanding of its pathogenesis.

However, most previous studies mainly focused on different gene expression patterns between the pSS and healthy subgroups, and there are less studies applying WGCNA to investigate the gene expression signature of pSS. In our study, we first combined WGCNA analysis with the conjoint analysis of different gene expression patterns, based on RNA sequencing and microarray datasets, to explore hub genes in SGs of pSS. The integrative study was followed by clinical validation. We were convinced that the study would help in the identification of biomarkers of pSS for early diagnosis, thus improving the prognosis and facilitating understanding of the underlying mechanisms of the disease.

## Materials and Methods

### Patients and Data Preparation

A total of 16 pSS patients and 13 patients in the non-pSS subgroup were recruited from the First Affiliated Hospital of Wenzhou Medical University to conduct RNA sequencing with salivary glands. For further validation of candidate gene expression in peripheral bloods, the peripheral blood samples of different groups (including 35 pSS, 20 non-pSS, 30 RA, and 23 systemic lupus erythematosus (SLE) subgroups) were also recruited from this hospital to perform quantitative reverse transcription polymerase chain reaction (RT-qPCR) experiments. In addition, another 58 pSS were recruited in our study, and the expression levels of the hub genes in their SGs were investigated by RT-qPCR to explore their relation with disease activity. The pSS patients fulfilled the 2016 American College of Rheumatology (ACR)/European League Against Rheumatism (EULAR) classification criteria (Shiboski et al., 2017) or 2012 ACR criteria (Shiboski et al., 2012). The RA patients fulfilled the 2010 ACR/ELUAR criteria (Aletaha et al., 2010), and the SLE patients were in accord with the 2019 ELUAR/ACR classification criteria (Aringer et al., 2019). The non-pSS subgroups were those who experienced subjective clinical symptoms of xerostomia or xerophthalmia but did not meet the classification criteria of pSS. All SG samples were conserved in RNAlater^®^ within −80°C for subsequent RNA sequencing, and the peripheral blood samples were also kept in −80°C after centrifuging at 1,000 revolutions/min for 10 min, prepared for subsequent quantitative reverse transcription polymerase chain reaction (RT-qPCR) analysis and enzyme-linked immunosorbent assay (ELISA) experiments. This study was approved by the Ethics Committee of the First Affiliated Hospital of Wenzhou Medical University, and written informed consent was received from all participants for their enrollment.

To increase the accuracy and reliability of the findings, we also downloaded four eligible microarray datasets from GEO with the following selection criteria: (a) inclusion of gene expression data of pSS, non-pSS, or healthy donors, excluding Sjogren syndrome cases which were associated with other autoimmune diseases such as RA, SLE, and so on, (b) using SG samples for microarray analysis rather than blood samples, and the patients had not received pSS systemic treatment before the salivary gland biopsy, and (c) inclusion of > 5,000 genes in every GEO platform.

### RNA Sequencing and Data Preprocessing

Total RNA from frozen SG samples were isolated using TRIzol^®^ Reagent (Invitrogen), and RNA purity was checked using the Nano Photometer^®^ spectrophotometer (IMPLEN, CA, United States). The high-quality of RNA [RNA integrity numbers (RIN) > 9] for cDNA library preparations was assessed by Bioanalyzer 2100 system using the Agilent RNA 6000 Nano kit (Agilent Technologies, CA, United States). Sequencing libraries were constructed using NEBNext Ultra^TM^ RNA Library Prep Kit for Illumina (NEB, United States), with the input material of 3 μg of RNA per sample, and the prepared libraries were subsequently sequenced on an Illumina HiSeq platform. Then, raw reads were trimmed using Cutadapt adapters, and low-quality reads were filtered using Trim Galore. Quality control reports of sequence reads were obtained through FastQC software^1^. Finally, the sequencing data wee aligned to the human reference genome hg38 using STAR software. The read count files were filtered with low expression and normalized by DEseq2 package (Love et al., 2014).

Four gene expression profiles were downloaded from GEO, and the k-nearest neighboring (KNN) imputation algorithm was conducted to impute the few missing values through the impute package (Hastie et al., 2001). Then, probes with zero (the lowest expression) were eliminated by a filtering process, and the ComBat method of R package sva (Leek et al., 2012) was used to remove known batch effects from microarray data. Finally, quantile normalization was conducted using normalizeWithinArrays and normalizeBetweenArrays functions, and the probe IDs were converted into gene symbols based on the annotation file for probes of the platform.

### Identification of Differentially Expressed Genes

We used princomp function to conduct a two-dimensional principal component analysis (PCA) and hierarchical clustering to visualize the similarities and the differences between the pSS and the non-pSS subgroups. Subsequently, the limma (Ritchie et al., 2015) and DEseq2 packages were used to screen the differentially expressed genes (DEGs) of microarray and RNA-seq data. The DEGs were identified based on the following criteria: adjusted *p* < 0.05 and absolute value of log2 fold change (FC) > 1. All the DEGs were visualized in three volcano plots using EnhancedVolcano package^2^, and the common DEGs were exhibited by clustering heat map and Venn diagram.

### Weighted Gene Co-expression Network Analysis

We extracted the top 25% genes with highest variance in ANOVA to construct the co-expression network using RNA-seq data with complete clinical information by the R package WGCNA (Langfelder and Horvath, 2008). Subsequently, the adjacency matrix was transformed into topological overlap matrix (TOM), and different gene modules were identified based on hierarchically clustering genes through TOM. Here the soft-thresholding power was set as 24 when 0.8 was used as the scale-free *R*^2^ threshold, and the minimum number of genes in the modules was set as 30. Moreover, the cut height threshold was set as 0.25 to merge possibly similar modules. The module that highly correlated with clinical phenotype was identified to conduct Kyoto Encyclopedia of Genes and Genomes (KEGG) pathway analysis, and hub genes were defined with gene significance (GS) > 0.5 and module membership (MM) > 0.9. Combined with the previously found common DEGs, common hub genes were ultimately identified for validation.

### Visualization of Chromosome Locations and Function Enrichment Analyses

To acquire detailed chromosome position information of the common DEGs, we downloaded gtf annotation of human (hg38) RefSeq transcripts from UCSC Genome Browser dataset^3^. Circos plots were created with function of RCircos.Gene.Connector.Plot and RCircos.Heatmap.Plot in RCircos package (Zhang et al., 2013). Correlation among the common DEGs was calculated by Pearson’s test through ggcorrplot package. For function enrichment analyses, Gene Ontology (GO) and KEGG pathway analyses were conducted by using ClusterProfiler package (Yu et al., 2012). The top two GO and all KEGG terms with adjusted *p* < 0.05 were visualized graphically by GOplot package (Walter et al., 2015) and “ClueGO” plugin in Cytoscape software, respectively (Bindea et al., 2009). The protein–protein interaction (PPI) networks of the common DEGs were downloaded from STRING database (Szklarczyk et al., 2011) and structured by Cytoscape.

### Single-Gene GSEA

To validate the function of *ICOS* in pSS, we divided the pSS subgroups into two groups with high or low expression levels of *ICOS* based on candidate gene scores, calculated as described in other studies (Kirou et al., 2004, 2005). The mean and SD level of *ICOS* in the non-pSS [mean (control) and SD (control)] were calculated to standardize the expression of *ICOS* for each sample. Then, the standardized expression levels of each patient were reckoned as per the following calculation formula:

$$\text{Candidate}-\mathrm{Gene}\,\mathrm{Sscores}\,\left(I\,C\,O\,S\right)\,\,i=\frac{I\,C\,O\,S\,\,i\,\mathrm{pss}-\text{Mean}\,\left(\text{control}\right)}{\text{SD}\,\left(\text{control}\right)},$$

where *i* = number of the patients and *ICOS i* pss = expression levels of *ICOS* in each pSS patient. Subsequently, the threshold of candidate gene scores was identified through double normal distribution model using mixtools package. The software of GESA v4.0 was used for GSEA of *ICOS* in pSS.

### Immune Infiltration Analysis

To further evaluate the immune cell infiltration features of SGs in pSS, we applied the Immune Cell Abundance Identifier (ImmuCellAI) to transform the gene expression profiles into immune infiltration files based on the abundance of 24 immune cell types^4^ (Miao et al., 2020). The heat map of immune infiltration was constructed using “pheatmap” R package, and the comparison of different immune cells among subgroups with different extents of immune infiltration scores was performed using Wilcoxon test. Moreover, the correction between the expression of *ICOS* and the infiltration scores of immune cells was conducted using “Spearman” methods.

### Phenotype Analysis of *ICOS* and Diagnostic Model for pSS

To understand the expression of *ICOS* in SGs and peripheral bloods of healthy cohorts, we downloaded RNA-seq data of *ICOS* in various tissues from GTEX database (GTEx Consortium, 2013), and the expression levels of *ICOS* were shown in violin plots. In addition, we divided the pSS and the non-pSS into opposing groups based on clinical phenotype to further evaluate the expression of *ICOS* with Wilcoxon test. Moreover, to further explore the correlation between the expression of *ICOS* and systematic involvements of pSS, we used EULAR Sjogren’s Syndrome Disease Activity Index (ESSDAI) scores to reflect the disease activity and systemic involvement of the participant (Seror et al., 2010) and then performed the correlation analysis between the expression of *ICOS* and clinical features. The randomForest package was applied to conduct random forest (RF) models with 100 runs of cross-validation, which predicted pSS based on *ICOS* gene expression and other clinical features, including focus score, anti-SSA/Ro positivity, and hypergammaglobulinemia. The ROCR package (Sing et al., 2005) was used to compute receiver operating characteristic (ROC) curves, and the ggplot2 package was applied to present the mean decrease accuracy and the mean decrease Gini to assess the impact of each variable in RF.

### RT-qPCR Analysis and ELISA Experiment Validation

Total RNA was isolated from peripheral blood samples using TRIzol^®^ reagent (Invitrogen) according to protocol, and 1 μg of total RNA was used for the reverse transcription and qPCR using the GoTaq^®^ 2-Step RT-qPCR System (Promega). *ICOS* gene of pSS was, respectively, assayed by qPCR on Applied Biosystems Real Time PCR Instrument (ABI) with three steps. For each PCR detection, after enzyme activation at 95°C for 2 min, amplification of 95°C was performed for 40 cycles and completed after 60°C for 60 s. For each example, the PCR was repeated three times, and the gene expression of *ICOS* was measured according to comparative ΔCt (ΔΔCt) method.

Fresh plasma was gained from peripheral blood samples of pSS after centrifugation at 2,400 rpm for 20 min using a centrifuge 5810^®^ (Eppendorf, Germany), and ELISA 96-well plate kits of six proteins (ICOS, IL-17A, IFN-, TGFβ1, IL-6, and IL-4) were used as the carrier with prepackaged enzyme-labeled antibody. Subsequently, 150 μl stock solution was serially diluted into standard dilutions with different concentrations (120, 60, 30, 15, and 7.5 ng/ml) to draw standard curves. Then, 50 μl plasma and 50 μl biotinylated antigen working solution were, respectively, added into each well and incubated at 37°C for 60 min. Following washing for five times, 50 μl avidin-HRP was added into the wells and incubated again at 37°C for 30 min. After reduplicated washing, 50 μl of chromogenic reagents A and B was used to develop the stain for 10 min, and 50 μl stop buffer was employed to stop the reaction. Finally, the absorbance of each well was measured at 450 nm using a Varioskan Flash (Thermo Fisher Scientific, United States), and the concentration of samples was calculated *via* “ELISAcalc” software with logistic model based on the standard curve.

### Statistical Analysis

The relative expression levels of the hub genes detected by RT-qPCR and the concentrations of these proteins in plasma detected by ELISA were presented as mean ± standard deviation, and a comparison among groups was performed using Wilcoxon test. *P* < 0.05 was considered as statistically significant.

## Results

### The Identification of DEGs in Subgroups of pSS

**FIGURE 1:**
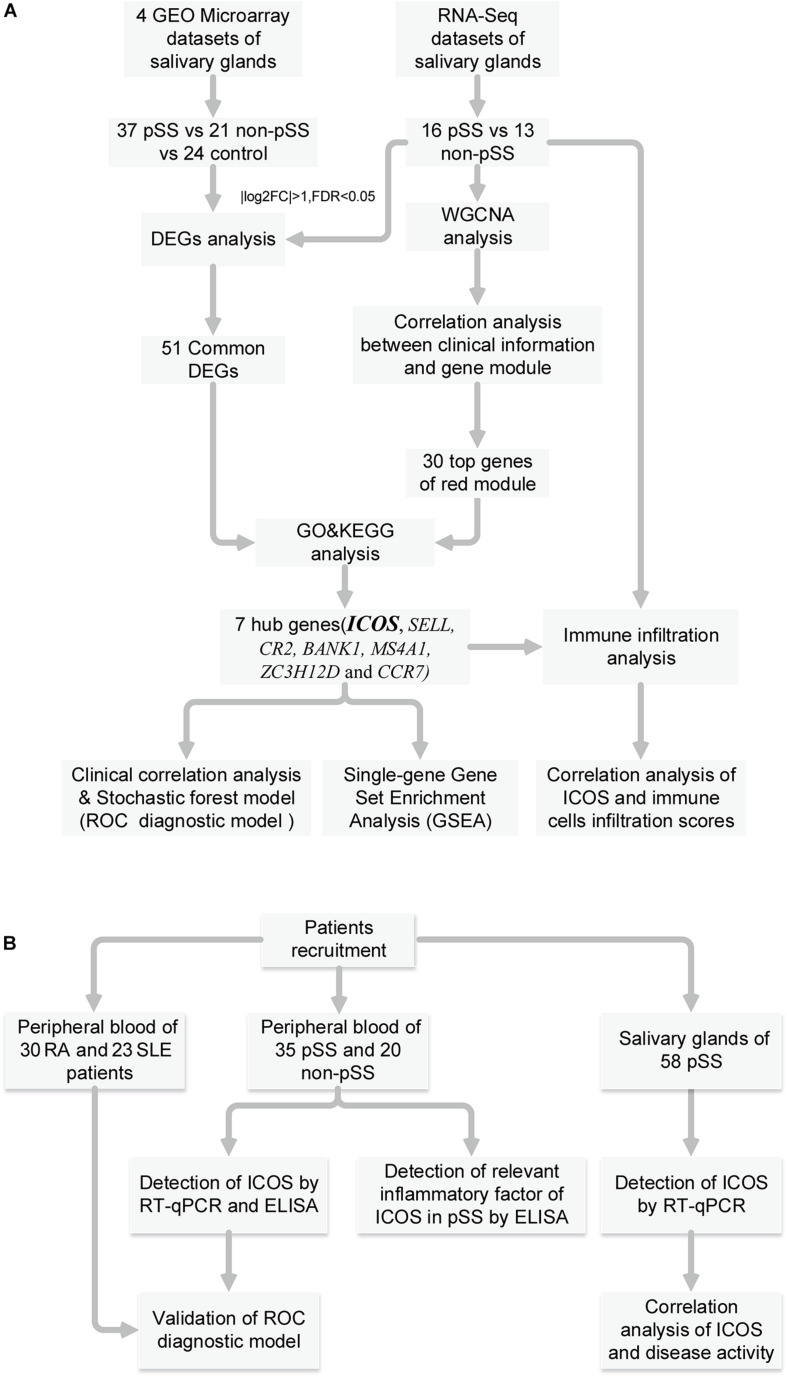
Summary and description of the study workflow. **(A)** The workflow of identification of hub genes, correlation analysis and construction of diagnostic model. **(B)** The workflow of special assessment of models and experimental validation for pSS.

**FIGURE 2:**
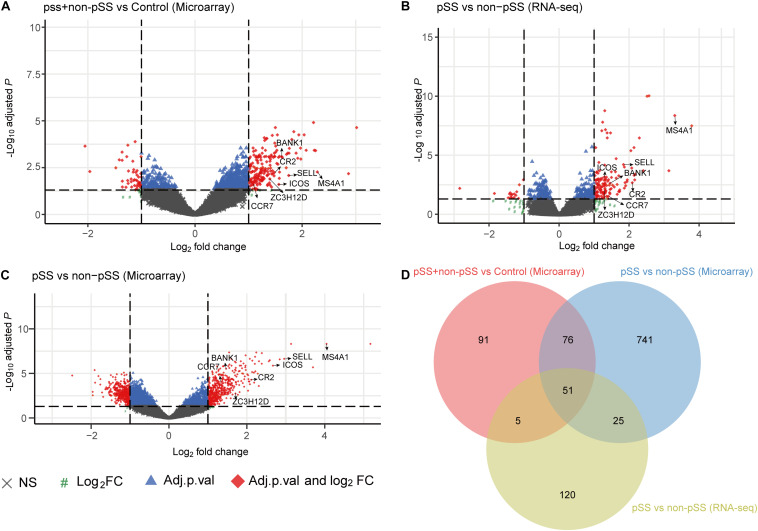
Volcano plot showing all the gene expression changes in primary Sjögren’s syndrome (pSS): **(A)** Differentially expressed genes (DEGs) of pSS + non-pSS vs. control in microarray data, **(B)** DEGs of pSS vs. non-pSS in RNA-seq data, **(C)** DEGs of pSS vs. non-pSS in microarray data, **(D)** Venn diagram showing the common DEGs of the abovementioned three comparisons.

Figure 1 shows the workflow of the whole process of our study. In accordance with the selection criteria, five eligible microarray datasets were chosen to preprocess and merge for subsequent variation analysis. The main characteristics of datasets are shown in Supplementary Table 1, and quality control showed that the gene expression distribution of each sample from the different resources were homogeneous and comparable after adjusting the batch effect (Supplementary Figures 1A,B). PCA analysis revealed that the pSS samples and the controls were generally separated into two distinct clusters (Supplementary Figures 1D,E), indicating the discriminative gene expression pattern of pSS. Based on the cutoff criteria [adjusted *p* < 0.05 and (log_2_ FC) > 1], a total of 127 DEGs were identified, including 120 up-regulated and seven down-regulated genes from microarray datasets (Figures 2A,C).

To further identify the changes of gene expression in the progress of pSS, we investigated the gene expression profiles of labial glands from 16 clinically definite pSS patients and 13 non-pSS patients by using RNA sequencing. The clinical characteristics of study cohorts are shown in Table 1, and the results displayed that the cohorts of pSS were homogeneous with low disease activity and rare systematic involvements (Supplementary Table 2). The PCA analysis revealed that the pSS and the non-pSS groups were clearly separated into two distinct clusters with obvious spatial separation (Supplementary Figure 1C). Subsequently, 180 up-regulated and 21 down-regulated genes were identified (Figure 2B), and 51 up-regulated common genes were chosen for subsequent analysis (Figure 2D). A list of 51 common DEGs with adjusted *p*-value is presented in Supplementary Table 3, and their gene expression pattern could obviously separate pSS (Supplementary Figure 1F).

**TABLE 1:** Clinical information of 16 primary Sjögren’s syndrome (pSS) and 13 non-pSS patients.

Characteristic	pSS (*n* = 16)	Non-pSS (*n* = 13)	*p*-value
Age (year)	50.31 ± 15.49	49.05 ± 11.96	0.630
Xerostomia, *n* (%)	8 (50%)	5 (38.46%)	0.710
Xerophthalmia, *n* (%)	5 (31.25%)	5 (38.46%)	0.714
IgG, g/L	19.27 ± 6.11	14.68 ± 4.15	0.025*
ESR, mm/H	28.88 ± 18.27	22.77 ± 20.51	0.150
ANA-positive (ANA > 1:100), *n* (%)	16 (100%)	9 (69.23%)	0.030*
Anti-SSA/Ro60-positive, *n* (%)	12 (75.00%)	5 (38.46%)	0.067
Anti-SSA/Ro52-positive, *n* (%)	12 (75.00%)	5 (38.46%)	0.067
Anti-La/SSB-positive, *n* (%)	12 (75.00%)	2 (15.38%)	0.003**
Focus score ≥ 1, *n* (%)	15 (93.75%)	1 (7.69%)	0.000***
ESSDAI score, median (Q1–Q3)	2 (1–4)	–	–

Note. IgG, Immunoglobulin G; ESR, erythrocyte sedimentation rate; *p < 0.05; **p < 0.01; ***p < 0.001.

### WGCNA and Identification of the Hub Genes

**FIGURE 3:**
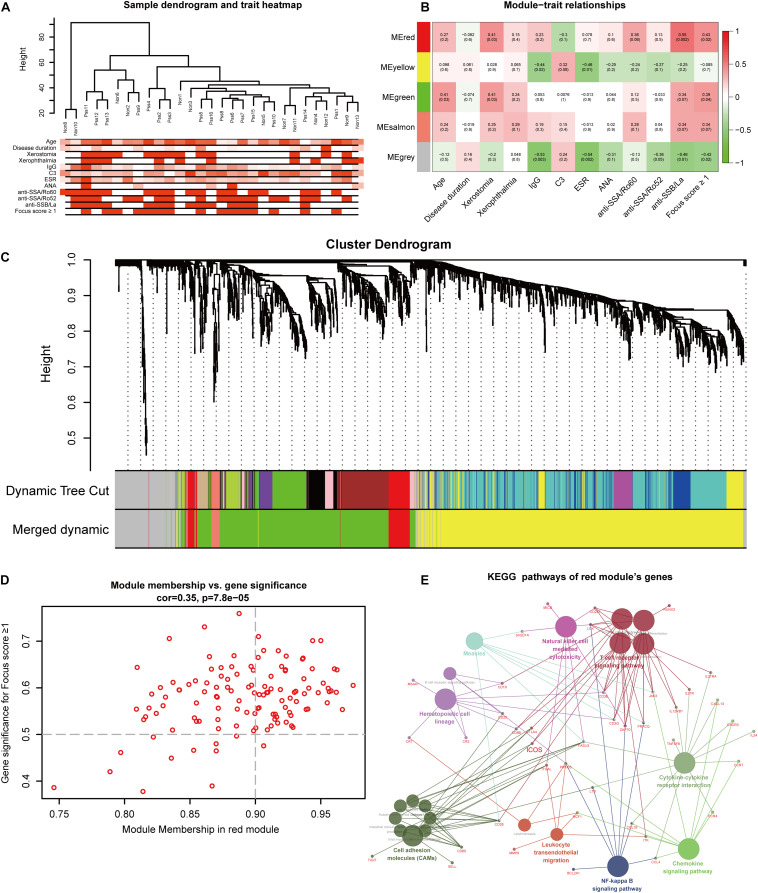
Identification of modules and hub genes closely associated with clinical traits of primary Sjögren’s syndrome (pSS) in RNA-seq data through weighted gene co-expression network analysis. **(A)** Hierarchical clustering dendrograms of genes with clinical traits, **(B)** heat map of the correlation between module eigenvalues and clinical traits of pSS containing the correlation coefficient and *p-*value, and **(C)** dendrogram of all genes showing the change of modules before and after merging. **(D)** Scatter plot of modular genes in the red module. **(E)** The network of Kyoto Encyclopedia of Genes and Genomes pathway in the red module.

To find the key modules most associated with pSS clinical traits, we performed WGCNA on the data of RNA sequencing. Clinical information of pSS including demographic (age and disease duration), symptomatic (xerostomia and xerophthalmia), serological [immunoglobulin G (IgG), complement 3 (C3), ESR, anti-nuclear antibody (ANA), anti-SSA/Ro, and anti-SSB/La], and histological features (focus score ≥ 1) are clustered in Figure 3A. By setting the soft-thresholding power as 24 (scale-free *R*^2^ = 0.8) and cut height as 0.25, we eventually identified five modules (Supplementary Figures 2A,B and Figure 3C; non-clustering genes shown in gray). From the heat map of module–trait correlations, we identified that the red module was most highly correlated with clinical traits (Figure 3B), especially with anti-SSB/La (correlation coefficient = 0.55, *p* = 0.002) and focus score ≥ 1 (correlation coefficient = 0.43, *p* = 0.02). To further investigate the correlation between MM in red module and GS for focus score ≥ 1, correlation analysis and clustering algorithm were performed. The results manifested that the red module contained a total of 122 genes (correlation coefficient = 0.35, *p* = 7.8e–05, Figure 3D) and the red module was highly associated with focus score ≥ 1 (Supplementary Figure 2C). In addition, the heat map of random 1,000 genes also showed the interrelation and stability of five modules (Supplementary Figure 2D), and KEGG analysis indicated that the red module significantly enriched in biological processes associated with inflammation including chemokine signaling pathway, cytokine–cytokine receptor interaction, T cell receptor signaling pathway, natural killer cell-mediated cytotoxicity, and cell adhesion molecules (CAMs) (Figure 3E). Moreover, 30 hub genes were chosen to manifest the module’s characteristic according to the chosen criterion with high GS and MM value. Combined with 51 common DEGs, seven hub genes (*ICOS, SELL*, *CR2*, *BANK1*, *MS4A1*, *ZC3H12D*, and *CCR7*) were identified for further analysis in Figure 5A.

### Visualization of DEGs’ Chromosome Locations and Characteristics

The 51 common DEGs were chosen to visualize their expression characteristics and chromosomal locations from hg38 dataset of Ensemble (Supplementary Figure 3). The results revealed that these DEGs were distributed in most chromosomes, except for chromosome X and Y. In addition, chromosomes 1, 4, and 6 contained most DEGs, and the top five genes according to adjusted *p-*value (*CXCL9*, *CXCL10*, *TAP1*, *MS4A1*, and *CXCL13*) were distributed in chromosomes 4, 6, and 11. Interestingly, the hub genes were mapped in chromosomes1, 2, 4, 6, 11, and 17. Notably, *ICOS* was adjacent to the acknowledged virulence gene *STAT1*, which participated in the pathway of activation of type I interferon (*IFN*), and *BANK1* was adjacent to the location of the C-X-C motif chemokine family.

### Functional Enrichment Analysis of DEGs

**FIGURE 4:**
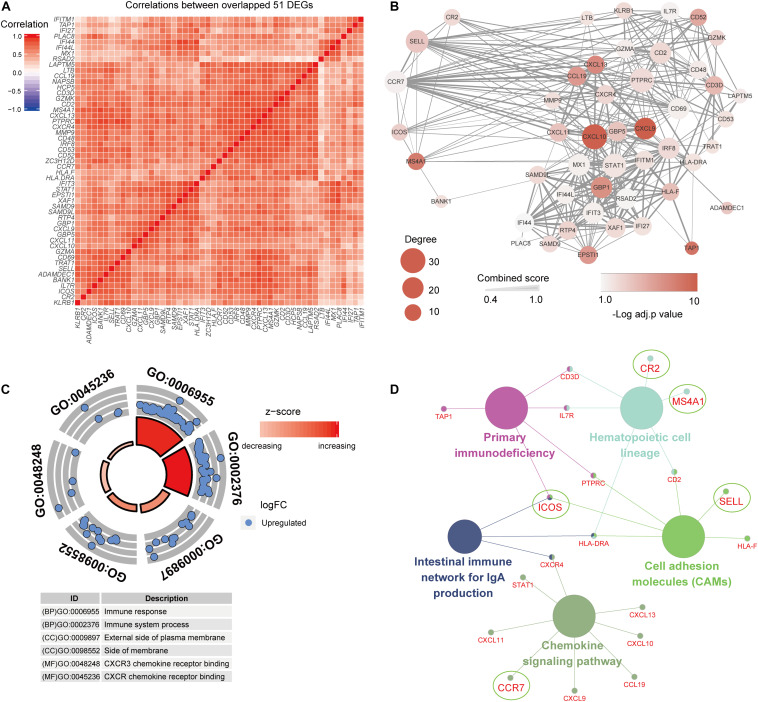
Functional enrichment analysis of 51 common differentially expressed genes (DEGs). **(A)** The common DEGs show strong associations with each other. **(B)** The PPI network shows a close interaction of DEGs, and hub genes indicate important roles in the network. **(C,D)** Gene Ontology and Kyoto Encyclopedia of Genes and Genomes pathway analyses of the common DEGs.

In order to further interpret biological processes associated with the gene signature of pSS, the 51 common DEGs were chosen to conduct GO function and KEGG pathway enrichment analysis based on significant correlation with each other (Figure 4A). It turned out 71 biological processes (BP), four cell components (CC), and nine molecular functions (MF) GO terms in Supplementary Table 4, and the DEGs were significantly enriched in processes such as immune response in BP, CXCR chemokine receptor binding in MF, and external side of plasma membrane in CC (Figure 4C). As to KEGG pathway analysis, five pathways (Supplementary Table 5) were identified, and an interaction relationship network of pathways comprised of DEGs is exhibited in Figure 4D. Interestingly, five hub genes (*ICOS, SELL*, *MS4A1*, *CR2*, and *CCR7*) also participated in those pathways, and *ICOS* was identified as a key molecule for subsequent validation and analysis based on its central node role in three pathways including CAMs, intestinal immune network for IgA production, and primary immunodeficiency. In addition, to evaluate the interaction of proteins of these DEGs, the PPI analysis showed an interconnected network with 49 gene nodes and 334 edges, of which the hub genes, especially *SELL, CCR7*, and *ICOS*, demonstrate core positions with a high degree of relatedness (Figure 4B).

**FIGURE 5:**
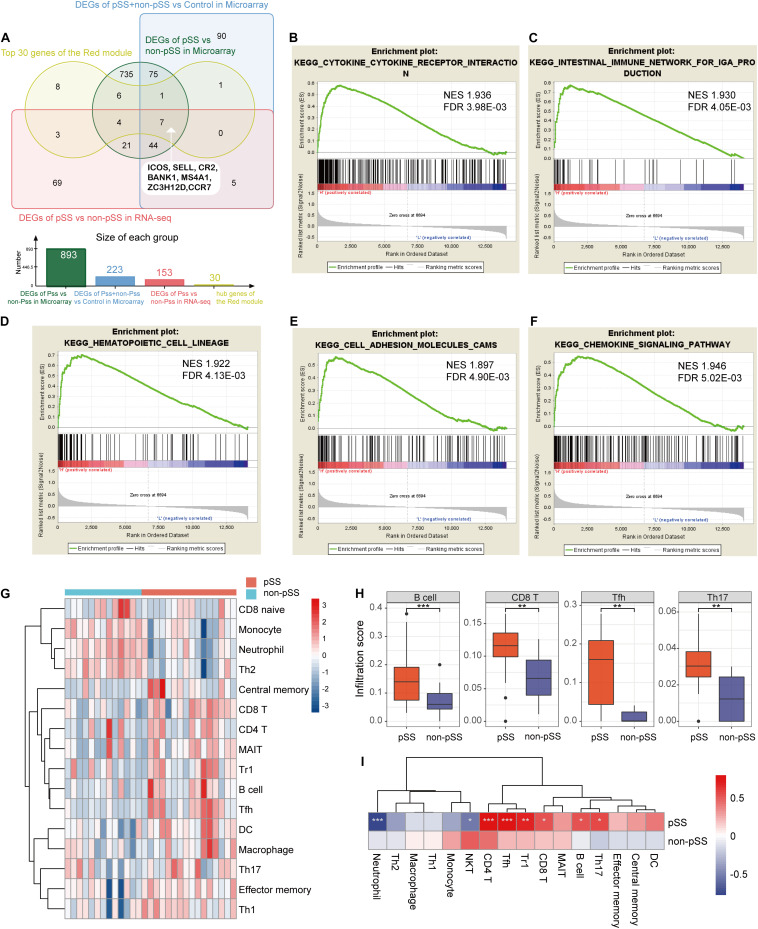
The results of single-gene Gene Set Enrichment Analysis based on the expression of *ICOS* and immune cell infiltration. **(A)** Venn diagram showing the identification of seven hub genes combining common differentially expressed genes (DEGs) and the red module. **(B–F)** The elevated *ICOS* expression was enriched in cytokine–cytokine receptor interaction **(B)**, intestinal immune network for IgA production **(C)**, hematopoietic cell lineage **(D)**, cell adhesion molecules **(E)**, and chemokine signaling pathway **(F)**. **(G)** Heat map showing the different infiltration degrees of 24 immune cells in primary Sjögren’s syndrome (pSS) and non-pSS with infiltration scores. **(H)** Box plots showing the most significant difference of infiltration scores in B cells, CD8^+^ T cells, Tfh cells, and Th17 cells between pSS and non-pSS. **(I)** Heat map displaying the correlation of *ICOS*’s expression and infiltration scores. *p < 0.05; **p < 0.01; ***p < 0.001.

### Single-Gene GSEA Reveal a Close Relationship Between *ICOS* and Inflammation

To find out significant pathways between low expression and high expression groups of *ICOS* and further investigate the potential functions of *ICOS* in pSS, we divided pSS and non-pSS patients into high and low subgroups based on double normal distribution curve and performed single-gene GSEA analysis using GSEA v4.0 software. Interestingly, the subsequent single-gene GSEA analysis confirmed that a low expression of *ICOS* was not enriched in any pathways, while a high expression of *ICOS* was linked to pathways of immunological activation including 20 inflammatory pathways (Supplementary Table 6). The top five pathways, including cytokine–cytokine receptor interaction, intestinal immune network for IgA production, hematopoietic cell lineage, CAMs, and chemokine signaling pathway, are exhibited in Figures 5B–F with significant *q* value and a normalized enrichment score, which consisted of the above preceding results.

### Immune Cell Infiltration Characterization in SGs of pSS

To present the features of immune cell infiltration in SGs in pSS, we conducted ImmuCellAI algorithm to compare the infiltration level of 24 immune cells in SGs using RNA-seq data. More significant immune cell infiltration exhibited in pSS cohorts compared with non-pSS group on the heat map (Figure 5G and Supplementary Table 7) and massive immune cells were found remarkably increased in pSS, including B cells, CD8^+^ T cells, Tfh cells, and Th17 cells (Figure 5H). Moreover, a correlation analysis between the expression of *ICOS* and the infiltration scores of immune cells revealed that the expression of *ICOS* was positively correlated to the infiltration scores of B cells, CD4^+^ T cells, CD8^+^ T cells, Th17 cells, Tfh cells, and Tr1 cells while negatively correlated to neutrophiles (Figure 5I).

### Clinical Interactions and Diagnostic Value of *ICOS* for pSS

To investigate the organizational expression specificity of *ICOS* in healthy individuals, gene expression profiles were generated based on the GTEx database. As shown in Supplementary Figure 4, we found that *ICOS* was over-expressed in several tissues such as spleen, lung, small intestine, and whole blood, while poorly expressed in normal minor salivary glands. These results suggested that the increased expression of *ICOS* in SGs is probably accompanying the progress of pSS. To further assess the interactions between *ICOS* and the clinical phenotype in pSS, we separated pSS and non-pSS cases into different groups according to the phenotypic terms and found that the expression levels of *ICOS* were higher in xerostomia-positive groups (*p* = 0.037), focus score ≥ 1 (*p* = 0.012), anti-SSA/Ro60-positive groups (*p* = 0.039) and high serum IgG groups (*p* = 0.039) than their corresponding groups (Supplementary Figures 5A–D), while no significant correlation was found in high serum ESR groups (*p* = 0.29), anti-SSA/Ro52-positive groups (*p* = 0.2), and xerophthalmia-positive groups (*p* = 0.7) (Supplementary Figures 5E–G). All these results showed that *ICOS* was closely associated with typical manifestations of pSS, indicating that *ICOS* might serve as potential biomarkers for the classification of pSS.

**FIGURE 6:**
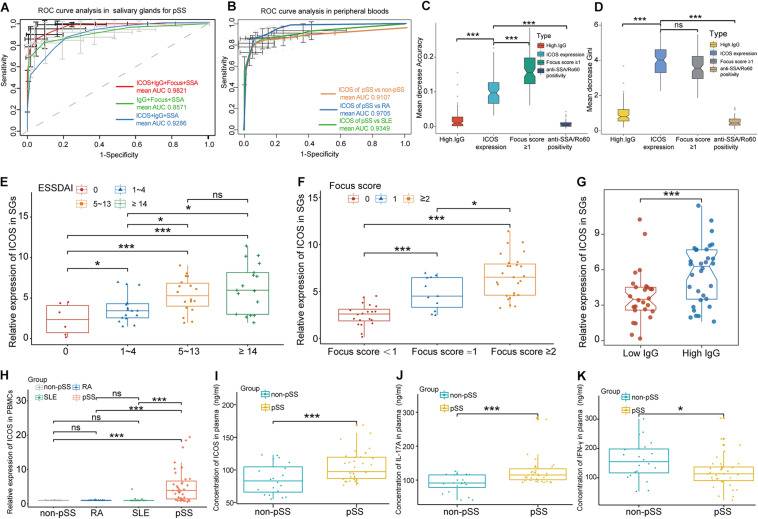
*ICOS* combined with traditional clinical indices for the diagnosis of primary Sjögren’s syndrome (pSS), clinical interaction analysis of *ICOS*, and experimental validation of *ICOS*. **(A)** Receiver operating characteristic curve of pSS prediction for the random forest (RF) model in salivary glands (SGs). The green lines indicate the diagnostic capacity of clinical indices without *ICOS* [are under the curve (AUC) 0.8571], the red lines indicate the diagnostic capacity of the model combining clinical indices and *ICOS* (AUC 0.9821), and the blue lines indicated the diagnostic capacity of the model clinical indices and *ICOS* without positive pathology (focus score ≥ 1). **(B)** ROC curve of pSS prediction for the RF model in peripheral blood using RT-qPCR. The orange lines indicate the diagnostic capacity of *ICOS* in peripheral blood for pSS vs. non-pSS with a high mean AUC value of 0.9107. The blue line and the green line, respectively, indicate the significant diagnostic capacity of ICOS in peripheral blood of pSS from rheumatoid arthritis (RA) (AUC 0.9705) and systemic lupus erythematosus (SLE) (AUC 0.9349). **(C,D)** Variable importance of *ICOS* and clinical variables of predicting pSS in salivary glands. Mean decrease accuracy represents the decrease of accuracy in the model when one variable is excluded, and mean decrease Gini represents the specific diagnostic capabilities of variables in the construction of the predicting model. **(E–G)** Violin plots showing the expression levels of *ICOS* in different subgroups based on ESSDAI scores **(E)**, focus scores **(F)**, and high IgG levels **(G)** in SGs. **(H)** The expression of *ICOS* on the aspect of gene was validated *via* RT-qPCR in the peripheral blood samples of non-pSS, RA, SLE, and pSS. **(I)** The expression of *ICOS* on the aspect of protein was validated *via* ELISA in peripheral blood samples of pSS. **(J,K)** Elevated IL-17A and decreased IFN-γ expressions in pSS were confirmed through ELISA experiment. *p < 0.05; **p < 0.01; ***p < 0.001.

To further evaluate the diagnostic value of *ICOS* in the diagnosis of pSS, ROC curve analysis was performed based on the expression levels of *ICOS* and typical characteristic indices of pSS, including focus score ≥ 1, anti-SSA/Ro60 positivity, and hypergammaglobulinemia. The combined model showed a high discriminatory accuracy in distinguished pSS from non-pSS, with a high mean AUC value of 0.9821 (Figure 6A). Moreover, the discriminant capability of the model was greatly reduced (with mean AUC value of 0.8571) if *ICOS* was excluded from the model, and the discriminant capability increased (with mean AUC value of 0.9286) when the pathology of SGs was replaced by the expression levels of *ICOS*. To further estimate the contribution of each index to the overall diagnostic value in pSS, RF were conducted by using ranking methods. Notably, although the mean decrease accuracy of up-regulated *ICOS* expression was lower than that of positive pathology, the mean decrease accuracy of *ICOS* in this model was more discriminatory than those of other clinical features (Figure 6C). Moreover, on the aspect of the mean decrease Gini, the contribution of *ICOS* was equal to the positive pathology, with a higher discriminatory power than others (Figure 6D).

To further investigate the correlation between grade of lymphocytic infiltration and the expression level of *ICOS*, another 58 pSS patients were included in our study and then divided into different groups based on lymphocytic infiltration in SGs. Their clinical characteristics are shown in Table 2. The expression of *ICOS* in SGs tested by RT-qPCR was found to be positively related to ESSDAI scores and positively associated with the grade of lymphocytic infiltration and hyperimmunoglobulin in pSS (Figures 6E–G), while there was no significant association was found with anti-SSA positivity, increased ESR, reduced C3, and keratoconjunctivitis sicca (Supplementary Figures 5H–K).

**TABLE 2:** Clinical information of 58 primary Sjögren’s syndrome patients.

Characteristic	Focus ≤ 1 (*n* = 31)	Focus ≥ 2 (*n* = 27)	*p*-value
Age (year)	51.87 ± 13.97	53.00 ± 13.74	0.920
Xerostomia, *n* (%)	15 (48.40%)	16 (59.30%)	0.408
Xerophthalmia, *n* (%)	20 (64.50%)	20 (74.10%)	0.433
IgG, g/L	16.63 ± 4.50	19.51 ± 6.30	0.011*
ESR, mm/H	25.52 ± 21.70	27.59 ± 24.10	0.810
ANA-positive (ANA > 1:100), *n* (%)	15 (48.40%)	18 (66.70%)	0.161
Anti-SSA/Ro60-positive, *n* (%)	22 (70.97%)	22 (81.48%)	0.351
Anti-SSA/Ro52-positive, *n* (%)	23 (74.20%)	23 (85.20%)	0.303
Anti-La/SSB-positive, *n* (%)	8 (25.80%)	10 (37.00%)	0.356
ESSDAI score, median (Q1–Q3)	4 (1–8)	9 (5–19)	0.017*

Note. IgG, Immunoglobulin G; ESR, erythrocyte sedimentation rate; *p < 0.05; **p < 0.01; ***p < 0.001.

### Experimental Validation of *ICOS* and Specificity for Diagnosis

To further validate *ICOS* mRNA expression profile in peripheral blood and the specificity of *ICOS* in the diagnosis for pSS, we performed RT-qPCR and ELISA using 35 pSS and 20 paired non-pSS samples. It revealed dramatically increased *ICOS* gene and protein expression in the whole blood of pSS than the non-pSS (Figures 6H,I), and *ICOS* also showed a high discriminatory accuracy for pSS in peripheral blood, with a high mean AUC value of 0.9107 (Figure 6B), through RT-qPCR. More importantly, the optimal cutoff value of *ICOS* expression was identified as 4.697 in SGs and 1.208 in PBMC to better discriminate pSS patients from non-pSS subgroups with high sensitivity and specificity (Table 3). Furthermore, to validate the special expression of *ICOS* in pSS, the peripheral blood samples of 30 RA and 23 SLE patients were used to perform RT-qPCR and to further conduct ROC analysis. The results demonstrated *ICOS*’s significant discriminative capacity between pSS and RA or SLE, with mean AUC values of 0.9705 and 0.9349 (Figure 6B), and the expression levels of ICOS in RA and SLE were almost close to that of non-pSS while significantly lower than that of pSS (Figure 6H). These results implied that ICOS was over-expressed in the PBMC of pSS and can serve as a potential biomarker for the classification of pSS with adequate sensitivity and specificity. To investigate the downstream protein regulated by *ICOS* in the mechanism of pSS, we also performed ELISA to detect the expression levels of inflammatory factors associated with *ICOS*, including *IL-6*, *IL-17A*, *TGF*β*1*, *IL-4*, and *IFN-*γ. Significantly, the expression of *IL-17A* (*p* = 0.00012) distinctly increased and that of *IFN-*γ (*p* = 0.02) decreased in peripheral blood of pSS (Figures 6J,K), while there was no difference in *TGF*β*1*, *IL-6*, and *IL-4* (Supplementary Figures 5L–N).

**TABLE 3:** Diagnostic value of *ICOS* in salivary glands (SGs) and peripheral blood mononuclear cells (PBMCs) for primary Sjögren’s syndrome (pSS).

Characteristic	Optimal cutoff point	Area under curve	Sensitivity	Specificity
**pSS vs. non-pSS**				
*ICOS* + focus + IgG + SSA (*n* = 29)	0.225	0.9821	0.875	0.975
*ICOS* + IgG + SSA (*n* = 29)	0.705	0.9286	0.954	0.857
*ICOS* in SGs (*n* = 29)	4.697	0.9330	0.875	0.923
*ICOS* in PBMCs (*n* = 55)	1.208	0.9107	0.771	0.914
**pSS vs. rheumatoid arthritis**				
*ICOS* in PBMCs (*n* = 65)	1.498	0.9705	0.974	0.743
**pSS vs. systemic lupus erythematosus**				
*ICOS* in PBMCs (*n* = 58)	1.587	0.9349	0.957	0.746

## Discussion

As a complex systemic autoimmune disease, the pathogenesis of pSS remains largely unclear. Although substantial biological molecule and genetic studies have been conducted to discover novel biomarkers and therapeutic targets for pSS, there is still a lack of explicit molecular mechanism and biological diadynamic criteria for pSS. As far as we know, our study is the first to apply WGCNA in gene expression profiles of pSS compared with non-pSS from GEO datasets and our RNA-seq profiles. We identified a total of 51 common robust DEGs, some of which, such as *EPSTI1*, *MMP9*, and *CXCL9*, have been reported to be biomarkers and participate in the pathogenesis of pSS (Song et al., 2014; Nezos et al., 2015). The chromosomal locations of 51 DEGs indicated that chromosome 1 contained the most DEGs, and Brayer’s study found that alleles on chromosome 1 and chromosome 3 may greatly influence the susceptibility and resistance to development of Sjögren’s syndrome-like autoimmune exocrinopathy in NOD mice models (Brayer et al., 2000). In addition, Pérez’s study reported *D1S3721* marker with significant differences in chromosome 1p34.2 with candidate genes (*LAPTM5*, *ZC3H12A*, and *NSAP*) in labial gland epithelial cell from pSS patients (Perez et al., 2009). Moreover, *STAT1* has been reported to be over-expressed in the labial salivary glands of pSS and associated with the pathway of activation of type I interferon (IFN) in pSS (Wakamatsu et al., 2006; Mavragani and Crow, 2010). All results suggested that these hub genes were located in key chromosomes with a potential influence of the pathogenesis of pSS.

Consistent with previously published studies, the enrichment of common DEGs in GO terms of our study, such as immune response, immune system process, cytokine-mediated signaling pathway, and cellular response to cytokine stimulus, further ensured their involvement in the progress of pSS (Routsias and Tzioufas, 2010; Song et al., 2014; Toro-Dominguez et al., 2014). Moreover, the KEGG pathway enrichment analysis of common DEGs also suggested their relevance in the pathogenesis of pSS. Chemokine signaling pathway was essential for maintaining the function and interaction of T lymphocytes (Ward and Westwick, 1998), and cytokine *CCL19* was identified as a biomarker of immunological activation in pSS with its chemokine receptor *CCR7* in our other study (Liu et al., 2019). It has been reported that adhesion molecules, such as intercellular adhesion molecule-1 and vascular cell adhesion molecule-1, were indispensable factors for lymphocyte recruitment, glandular damage, and the development of vasculitis in pSS, indicating the importance of CAM pathway in the mechanism of pSS (Turkcapar et al., 2005). Based on the results of GO and KEGG analyses, we confirm that these DEGs are closely associated with immune infiltration-related pathways and can serve as biomarkers for pSS.

In this study, co-expression network construction through WGCNA analysis obtained a total of five co-expression modules, and of them, the red module was the main one associated with positive pathology for pSS, containing 122 genes. KEGG pathway analysis of the red module was also enriched in the same pathways including chemokine signaling pathway, cytokine–cytokine receptor interaction, and CAMs, consisting of common DEGs. Moreover, it has been reported that the dysregulation of the *NF-kB* signaling pathway in B cell may alter the inflammatory response through regulating the expression levels of *BAFF* in pSS (Reksten et al., 2016), and the number and killing activity of natural killer (NK) cells decreased through natural killer cell-mediated cytotoxicity pathway in pSS (Izumi et al., 2006). All the results confirmed that the red module was closely associated with immune processes of pSS. After combining with the common DEGs, we eventually obtained seven hub genes (*ICOS*, *SELL*, *CR2*, *BANK1*, *MS4A1*, *ZC3H12D*, and *CCR7*) and identified *ICOS* to demonstrate its correlation with the pathogenesis of pSS.

Notably, most hub genes have been considered related to immunization and inflammation in autoimmune diseases. L-Selectin (*SELL/CD62L*), one of the adhesion molecules, has been extensively reported to be associated with lymphocytic infiltration, Raynaud’s phenomenon, and rheumatoid factor in pSS, but its concrete role in the mechanism of pSS remained unclear (Garcia-Carrasco et al., 2000). Membrane spanning 4-domains A1 (*MS4A1*), also called CD20, has been recognized as a significant marker of B cell. Lymphocytes involving B cells are major types of immune cells infiltrating the salivary glands of SS patients (Mariette and Criswell, 2018). In our study, massive B cells were found to be significantly infiltrated in the SGs of pSS by immune infiltration analysis, which is consistent with the high expression levels of *MS4A1.* In addition, complement receptor 2 (*CR2/CD21*) was located at follicular dendritic cell networks for the development of ectopic lymphoid structures in labial gland biopsies of patients with SS (Bombardieri and Pitzalis, 2012; Kurshumliu et al., 2019). Similarly, the branchpoint-site single-nucleotide polymorphisms rs17266594 and rs10516487 in the B cell scaffold protein with ankyrin repeats 1 (*BANK1*) gene have been testified to be associated with various autoimmune diseases including SLE (Guan et al., 2011), RA (Orozco et al., 2009), and SSc (Rueda et al., 2010). Moreover, in our other study, we had found that elevated *CCL19/CCR7* expression in the salivary gland associated with anti-SSA/Ro antibody and IgG levels in pSS patients could serve as markers of immunological activation in pSS (Liu et al., 2019). Interestingly, although the inducible costimulatory molecule (*ICOS*), as a member of the *CD28* family of coreceptor molecules, has been suggested to induce the difference of interleukin 17 (IL-17)-producing helper T cells (Th17 cells) and follicular helper T cells (Tfh cells), there are rare studies reporting the relativity between *ICOS* and pSS (Dong et al., 2001; Bauquet et al., 2009). Moreover, we also discovered a significant infiltration of Th17 cells and Tfh cells in SGs, and the expression of *ICOS* was significantly positively associated with their infiltration degrees, suggesting that *ICOS* might participate in the process of T cell activation in pSS.

In our study, clinical interactions analysis revealed that the expression of *ICOS* was significantly positively correlated with typical clinical characteristics of pSS, including lymphocytic infiltration in SGs, ESSDAI score, and hyperimmunoglobulin in pSS. The lymphocytic infiltration degree in SGs was placed as one of the most important indexes in the EULAR/ACR criteria for pSS (Shiboski et al., 2012, 2017). In addition, a correlation analysis of verification cohorts further identified that the expression of *ICOS* was highly positively associated with ESSDAI score and the grade of focus score, implying that *ICOS* might be associated with the disease activity of pSS. Furthermore, ROC curves showed that *ICOS* gene could serve as a biomarker to improve discrimination for pSS combined with traditional pathological and serologic indices, with a high mean AUC value of 0.9821 in SGs and 0.9107 in PBMC. Moreover, the results of our analysis using a ranking method with an RF model showed that *ICOS* gene was a significant index for the diagnosis of pSS, with greater discriminator capacity than other clinical and serologic features of pSS and without worse diagnostic performance than pathological positivity in mean decrease Gini. Besides significantly increasing in salivary glands, the expression of *ICOS* was also verified to be extremely increased on the aspect of gene and protein in peripheral blood samples by ELISA experiments. Previous genome-wide association studies had indicated common risk polymorphisms among RA, SLE, and pSS (Graham et al., 2007; Remmers et al., 2007). To raise the specificity of diagnosis for *ICOS* in pSS, the results of ROC analysis of RT-qPCR datasets of RA and SLE patients showed the expression of *ICOS* with preeminent discriminative capacity between pSS and RA/SLE. Therefore, *ICOS* may serve as an attractive target for the development of clinically useful biomarkers of pSS.

To further explore the biological functions of *ICOS* in the mechanism of pSS, we conducted GSEA using subgroups based on the expression of *ICOS*. The results of GSEA indicated that immune infiltration-related KEGG pathways such as CAMs, chemokine signaling pathway, and T cell receptor signaling pathway were enriched in the high-expression groups of *ICOS*, suggesting *ICOS*’s contribution to immune reaction in pSS. Moreover, ELISA experiments of inflammatory factors revealed *IL-17* with high expression and *IFN-*γ with low expression, while others (*IL-4*, *IL-6*, and *TGF-*β) were without significant difference in the peripheral blood of pSS in our study. Interestingly, Th17 cells have been considered as new CD4 helper T cell subsets that are essential in the pathogenesis of plenty of autoimmune diseases through animal models including RA, psoriasis, and multiple sclerosis (Matusevicius et al., 1999; Lock et al., 2002). Sakai et al. (2008) also found that salivary gland and acinar cells expressed IL-18R and IL-17R on the cell surface and IL-17 induced Th17 cells infiltrating in the salivary gland of pSS. Similarly, Li et al. (2012) also found that CD4(+) CXCR5(+) T cells (Th17-like subsets) played a significant role in pSS *via* efficiently inducing naïve B cells to produce immunoglobulin. More importantly, *ICOS* was found to regulate the differentiation of Th17 and Tfh cells through producing *IL-21*, *IL-17*, and *c-Maf* in Aurelie’s work, while Th17 cells have been identified to play an important role in immune response and auto-immune pathogenesis of pSS by zoopery (Lin et al., 2015; Matsui and Sano, 2017). Moreover, higher fractions of ICOS(+) Tfh cells were observed, and a positive association was found between autoantibody levels and increased level of Tfh cells in pSS compared to controls (Brokstad et al., 2018). Recent research also highlighted Tfh and pathogenic peripheral-helper T-cells (Tph), IL-21, and the ICOS costimulatory pathway as key pathogenic players in SS immunopathology (Pontarini et al., 2020). In addition, low IFN-γ levels and indifferent expression levels of other factors (*IL-6*, *IL-4*, and *TGF*β*1*) were found in ELISA, and this controversial finding may be explained by low disease activity in our pSS patients (Karabulut et al., 2018). Previous studies also found just a minor high expression of *IFN-*γ mRNA in salivary gland tissues in pSS, such as three out of 12 in Boumba’s work (Boumba et al., 1995) and 31 out of 53 in Hall’s study (Hall et al., 2015). Overall, the results suggested that *ICOS* may participate in the pathogenesis of pSS through regulating Th17 cells to produce IL-17, and IL-17 may serve as a promising therapeutic target for the biotherapy of pSS.

However, there still are some limitations in our study. On the one hand, although the study is the first one to conduct gene expression profiles using WGCNA combing chips with RNA-seq datasets, the small sample size is still limited, and thus there is a need for further studies to support our study. On the other hand, the diagnostic value of *ICOS* for pSS requires more congeneric researches, even clinical practices, to test and improve. In addition, the expression of *ICOS* in salivary gland from other etiologies (such as lymphomas, sarcoidosis, chronic sialadenitis, etc.) also needs to be tested to demonstrate its uniqueness in pSS. Furthermore, *ICOS* was found to be up-regulated both in SGs and PBMC, while its detailed role in the pathogenesis of pSS remains to be verified by in-depth *in vivo* and *in vitro* studies on molecular mechanism.

## Conclusion

In conclusion, we observed and validated a high prevalence of *ICOS* in both labial glands and peripheral bloods on the aspect of genes and proteins in pSS. Moreover, *ICOS* was found to be involved with the development of pSS through promoting immune infiltration such as regulating the differentiation of Th17 cells to produce IL-17. In addition, significantly elevated *ICOS* expression in pSS, which was correlated with ESSDAI scores, elevated IgG levels, and pathological infiltration levels in pSS, implicates a distinct discrimination for pSS. The various gene expression pattern analyses between pSS and non-pSS deepen our understanding of the disease mechanisms and suggest that *ICOS* is a promising biomarker for the detection of pSS.

## Data Availability Statement

The original contributions presented in the study are publicly available. This data can be found here: Gene Expression Omnibus (GEO) (https://www.ncbi.nlm.nih.gov/geo/) (Accessions: GSE159574). Publicly available datasets were analyzed in this study. This data can be found here: Gene Expression Omnibus (GEO) (https://www.ncbi.nlm.nih.gov/geo/) (Accessions: GSE40611, GSE127952, GSE23117 and GSE80805).

## Ethics Statement

The studies involving human participants were reviewed and approved by the Ethics Committee of the First Affiliated Hospital of Wenzhou Medical University. The patients/participants provided their written informed consent to participate in this study.

## Author Contributions

JL and XL contributed to data analysis and drafting of the manuscript. LZ and XX contributed to data analysis. SY contributed to data acquisition and figure presentations. MY and LXZ contributed to RT-qPCR and ELISA experiments. SL and XW contributed to the design of the study and revision of the manuscript. All authors contributed to the article and approved the submitted version.

## Conflict of Interest

The authors declare that the research was conducted in the absence of any commercial or financial relationships that could be construed as a potential conflict of interest.

## Footnotes

1 http://www.bioinformatics.babraham.ac.uk/projects/fastqc/

2 https://github.com/kevinblighe/EnhancedVolcano

3 http://genome.ucsc.edu

4 http://bioinfo.life.hust.edu.cn/web/ImmuCellAI
