# Supplementary material for: Transcriptome Sequencing Reveals Potential Roles of ICOS in Primary Sjögren’s Syndrome
Source: Front Cell Dev Biol. 2020 Dec 4;8:592490. doi: 10.3389/fcell.2020.592490 (PMC7747463; doi:10.3389/fcell.2020.592490)
Supplement: Supplementary file 1 [file Data_Sheet_1.zip › Supplementary Material/Supplementary Table.docx]

| \| **GEO ID** \| **pSS/non-pSS/Control** \| **Platform** \| **Sample source** \| **Country** \| **Years** \| **Reference (PMID)** \| \| --- \| --- \| --- \| --- \| --- \| --- \| --- \| \| GSE40611 \| 17/14/18 \| GPL570; [HG-U133_Plus_2] Affymetrix Human Genome U133 Plus 2.0 Array \| [Homo sapiens](https://www.ncbi.nlm.nih.gov/Taxonomy/Browser/wwwtax.cgi?mode=Info&id=9606) \| USA \| 2012 \| Horvath et al. (23116360) \| \| GSE127952 \| 8/0/6 \| [GPL2099](https://www.ncbi.nlm.nih.gov/geo/query/acc.cgi?acc=GPL6244)5; Agilent-019415 Human and Custom Viral Transcript Array 1.2 \| [Homo sapiens](https://www.ncbi.nlm.nih.gov/Taxonomy/Browser/wwwtax.cgi?mode=Info&id=9606) \| USA \| 2019 \| None \| \| GSE23117 \| 11/4/0 \| GPL570; [HG-U133_Plus_2] Affymetrix Human Genome U133 Plus 2.0 Array \| [Homo sapiens](https://www.ncbi.nlm.nih.gov/Taxonomy/Browser/wwwtax.cgi?mode=Info&id=9606) \| USA \| 2011 \| Greenwell-Wild et al. (21618203) \| \| GSE80805 \| 2/2/0 \| GPL13667; [HG-U219] Affymetrix Human Genome U219 Array \| [Homo sapiens](https://www.ncbi.nlm.nih.gov/Taxonomy/Browser/wwwtax.cgi?mode=Info&id=9606) \| Japan \| 2016 \| None \|   **Supplementary Table 1. Characteristics of the individual studies.** |
| --- | --- | --- | --- | --- | --- | --- | --- | --- | --- | --- | --- | --- | --- | --- | --- | --- | --- | --- | --- | --- | --- | --- | --- | --- | --- | --- | --- | --- | --- | --- | --- | --- | --- | --- | --- |

Supplementary Material

RA, rheumatoid arthritis; SLE, systemic lupus erythematosus; polyJIA, polyarticular type juvenile idiopathic arthritis; sJIA, systemic-onset JIA; HC, healthy children; HI, healthy individual.

**Supplementary Table 2. Clinical information of Discovery set (16 pSS and 13 non-pSS).**

| **Sample** | N6 | N2 | N13 | N5 | N11 | N1 | P10 | P4 | P14 | P16 | P8 | P15 | P7 | P2 | P9 | P1 | N8 | N12 | N9 | N3 | P11 | P12 | P5 | P6 | N10 | P13 | N4 | P3 | N7 |
| --- | --- | --- | --- | --- | --- | --- | --- | --- | --- | --- | --- | --- | --- | --- | --- | --- | --- | --- | --- | --- | --- | --- | --- | --- | --- | --- | --- | --- | --- |
| **Age** | 34 | 49 | 50 | 55 | 55 | 56 | 45 | 69 | 23 | 59 | 27 | 33 | 57 | 43 | 48 | 70 | 40 | 40 | 60 | 67 | 56 | 57 | 58 | 29 | 30 | 68 | 37 | 63 | 65 |
| **Disease duration** | 10 | 1 | 48 | 0 | 36 | 5 | 3 | 0 | 84 | 0 | 1 | 1 | 0 | 12 | 48 | 0 | 60 | 240 | 2 | 12 | 4 | 72 | 120 | 18 | 24 | 12 | 0 | 36 | 0 |
| **Xerostomia** | 1 | 0 | 0 | 0 | 1 | 1 | 0 | 1 | 0 | 1 | 0 | 0 | 0 | 1 | 0 | 0 | 0 | 1 | 1 | 0 | 1 | 1 | 1 | 0 | 0 | 1 | 0 | 1 | 0 |
| **Xerophthalmia** | 0 | 0 | 1 | 0 | 1 | 1 | 0 | 0 | 0 | 1 | 0 | 0 | 0 | 1 | 0 | 0 | 0 | 0 | 1 | 0 | 1 | 1 | 0 | 0 | 0 | 1 | 1 | 0 | 0 |
| **IgG** | 13.5 | 15.8 | 14.8 | 11.9 | 12 | 14.8 | 13.7 | 12.1 | 15.6 | 21.9 | 17.5 | 13 | 20.8 | 23.4 | 11 | 19.9 | 13.8 | 17 | 10.7 | 13.1 | 34.9 | 19.9 | 26.7 | 22.9 | 27.3 | 15.7 | 13 | 19.3 | 13.1 |
| **C3** | 0.94 | 0.98 | 0.97 | 1.38 | 1.23 | 0.94 | 1.02 | 1.04 | 0.98 | 1.19 | 1.23 | 0.9 | 1.05 | 1.01 | 0.73 | 1.32 | 1.07 | 0.71 | 1.36 | 1.39 | 0.92 | 0.81 | 0.95 | 1 | 0.85 | 0.93 | 0.96 | 0.73 | 1.19 |
| **ESR** | 11 | 60 | 7 | 10 | 10 | 10 | 11 | 15 | 22 | 29 | 18 | 26 | 28 | 22 | 9 | 39 | 13 | 8 | 57 | 34 | 88 | 25 | 41 | 23 | 50 | 28 | 2 | 38 | 24 |
| **ANA** | 0 | 100 | 1000 | 100 | 100 | 0 | 100 | 100 | 100 | 1000 | 100 | 100 | 100 | 100 | 1000 | 100 | 100 | 0 | 100 | 100 | 3200 | 100 | 100 | 3200 | 100 | 100 | 0 | 100 | 100 |
| **SSA** | 0 | 0 | 0 | 1 | 0 | 0 | 0 | 1 | 1 | 0 | 1 | 1 | 0 | 1 | 0 | 1 | 1 | 0 | 0 | 1 | 1 | 1 | 1 | 1 | 1 | 1 | 1 | 1 | 0 |
| **Ro52** | 0 | 1 | 0 | 0 | 0 | 0 | 1 | 0 | 1 | 0 | 1 | 1 | 1 | 1 | 1 | 0 | 0 | 0 | 1 | 0 | 1 | 1 | 1 | 1 | 1 | 0 | 1 | 1 | 1 |
| **SSB** | 0 | 0 | 0 | 0 | 0 | 0 | 0 | 1 | 1 | 1 | 0 | 1 | 1 | 1 | 0 | 0 | 0 | 0 | 0 | 1 | 1 | 1 | 1 | 1 | 1 | 1 | 0 | 1 | 0 |
| **Chisholm grade** | 1 | 1 | 1 | 1 | 1 | 1 | 2 | 3 | 4 | 1 | 3 | 4 | 1 | 2 | 2 | 3 | 3 | 3 | 3 | 3 | 1 | 1 | 1 | 3 | 4 | 3 | 4 | 4 | 4 |
| **Focus score** | 0 | 0 | 0 | 0 | 0 | 0 | 0 | 1 | 2 | 0 | 1 | 2 | 0 | 0 | 0 | 1 | 1 | 1 | 1 | 1 | 0 | 0 | 0 | 1 | 2 | 1 | 2 | 2 | 2 |
| **ESSDAI** | 2 | 0 | 0 | 7 | 0 | 0 | 0 | 0 | 0 | 1 | 1 | 1 | 2 | 2 | 3 | 3 | 0 | 1 | 0 | 0 | 4 | 4 | 4 | 4 | 4 | 4 | 1 | 15 | 5 |
| **Constitutional3** | 0 | 0 | 0 | 0 | 0 | 0 | 0 | 0 | 0 | 0 | 0 | 0 | 0 | 0 | 0 | 0 | 0 | 0 | 0 | 0 | 0 | 0 | 0 | 0 | 0 | 0 | 0 | 0 | 0 |
| **Lymphadenopathy4** | 0 | 0 | 0 | 0 | 0 | 0 | 0 | 0 | 0 | 0 | 0 | 0 | 0 | 0 | 0 | 0 | 0 | 0 | 0 | 0 | 0 | 0 | 0 | 0 | 0 | 0 | 0 | 0 | 0 |
| **Glandular2** | 0 | 0 | 0 | 0 | 0 | 0 | 0 | 0 | 0 | 0 | 0 | 0 | 0 | 0 | 0 | 0 | 0 | 0 | 0 | 0 | 0 | 0 | 0 | 0 | 0 | 0 | 0 | 0 | 0 |
| **Articular2** | 0 | 0 | 0 | 0 | 0 | 0 | 0 | 0 | 0 | 0 | 0 | 0 | 0 | 0 | 0 | 0 | 0 | 0 | 0 | 0 | 0 | 0 | 0 | 0 | 0 | 0 | 0 | 0 | 0 |
| **Cutaneous3** | 0 | 0 | 0 | 0 | 0 | 0 | 0 | 0 | 0 | 0 | 0 | 0 | 0 | 0 | 0 | 0 | 0 | 0 | 0 | 0 | 0 | 0 | 0 | 0 | 0 | 0 | 0 | 0 | 0 |
| **Pulmonary5** | 0 | 0 | 0 | 1 | 0 | 0 | 0 | 0 | 0 | 0 | 0 | 0 | 0 | 0 | 0 | 0 | 0 | 0 | 0 | 0 | 0 | 0 | 0 | 0 | 0 | 0 | 0 | 0 | 1 |
| **Renal5** | 0 | 0 | 0 | 0 | 0 | 0 | 0 | 0 | 0 | 0 | 0 | 0 | 0 | 0 | 0 | 0 | 0 | 0 | 0 | 0 | 0 | 0 | 0 | 0 | 0 | 0 | 0 | 2 | 0 |
| **Muscular6** | 0 | 0 | 0 | 0 | 0 | 0 | 0 | 0 | 0 | 0 | 0 | 0 | 0 | 0 | 0 | 0 | 0 | 0 | 0 | 0 | 0 | 0 | 0 | 0 | 0 | 0 | 0 | 0 | 0 |
| **PNS5** | 0 | 0 | 0 | 0 | 0 | 0 | 0 | 0 | 0 | 0 | 0 | 0 | 0 | 0 | 0 | 0 | 0 | 0 | 0 | 0 | 0 | 0 | 0 | 0 | 0 | 0 | 0 | 0 | 0 |
| **CNS5** | 0 | 0 | 0 | 0 | 0 | 0 | 0 | 0 | 0 | 0 | 0 | 0 | 0 | 0 | 0 | 0 | 0 | 0 | 0 | 0 | 0 | 0 | 0 | 0 | 0 | 0 | 0 | 0 | 0 |
| **Hematological2** | 1 | 0 | 0 | 1 | 0 | 0 | 0 | 0 | 0 | 0 | 0 | 0 | 0 | 0 | 1 | 1 | 0 | 0 | 0 | 0 | 1 | 1 | 1 | 1 | 1 | 1 | 0 | 2 | 0 |
| **Biological1** | 0 | 0 | 0 | 0 | 0 | 0 | 0 | 0 | 0 | 1 | 1 | 1 | 2 | 2 | 1 | 1 | 0 | 1 | 0 | 0 | 2 | 2 | 2 | 2 | 2 | 2 | 1 | 1 | 0 |

**Supplementary Table 3. List of the 51 common DEGs according to the rank of *adjust p value*.**

| **ID** | ***adj.p*** | **ID** | ***adj.p*** | **ID** | ***adj.p*** |
| --- | --- | --- | --- | --- | --- |
| CXCL9 | 9.80E-11 | RTP4 | 5.53E-04 | TRAT1 | 3.61E-03 |
| CXCL10 | 1.07E-10 | GZMK | 5.80E-04 | CD53 | 3.63E-03 |
| TAP1 | 1.80E-09 | CXCR4 | 8.02E-04 | NAPSB | 4.05E-03 |
| MS4A1 | 4.56E-09 | CD2 | 1.10E-03 | STAT1 | 4.66E-03 |
| CXCL13 | 3.39E-08 | XAF1 | 1.23E-03 | LAPTM5 | 7.92E-03 |
| GBP1 | 8.92E-08 | SAMD9L | 1.24E-03 | CD48 | 8.59E-03 |
| HCP5 | 1.36E-07 | CR2 | 1.29E-03 | ZC3H12D | 8.89E-03 |
| CCL19 | 3.60E-07 | KLRB1 | 1.29E-03 | GZMA | 1.29E-02 |
| EPSTI1 | 3.60E-07 | IRF8 | 1.29E-03 | MX1 | 1.42E-02 |
| CD52 | 2.38E-06 | SAMD9 | 1.31E-03 | CD69 | 1.46E-02 |
| CD3D | 2.06E-05 | PTPRC | 1.38E-03 | IFIT3 | 1.47E-02 |
| HLA-F | 4.29E-05 | MMP9 | 2.22E-03 | CCR7 | 1.77E-02 |
| CXCL11 | 6.49E-05 | BANK1 | 2.48E-03 | IFI44L | 2.12E-02 |
| GBP5 | 7.21E-05 | IFITM1 | 2.88E-03 | IL7R | 2.38E-02 |
| SELL | 1.05E-04 | IFI27 | 3.13E-03 | RSAD2 | 3.09E-02 |
| ADAMDEC1 | 1.27E-04 | HLA-DRA | 3.55E-03 | IFI44 | 4.51E-02 |
| ICOS | 2.66E-04 | LTB | 3.58E-03 | PLAC8 | 4.64E-02 |

**Supplementary Table 4. Results of GO function enrichment of 51 common DEGs**

| **Category** | **ID** | **GOTerm** | ***adj.p*** |
| --- | --- | --- | --- |
| BP | GO:0006955 | Immune response | 3.32E-22 |
| BP | GO:0002376 | Immune system process | 9.33E-21 |
| BP | GO:0051707 | Response to other organism | 5.98E-15 |
| BP | GO:0043207 | Response to external biotic stimulus | 6.19E-15 |
| BP | GO:0009607 | Response to biotic stimulus | 9.21E-15 |
| BP | GO:0019221 | Cytokine-mediated signaling pathway | 2.99E-14 |
| BP | GO:0071345 | Cellular response to cytokine stimulus | 1.94E-13 |
| BP | GO:0006952 | Defense response | 2.23E-13 |
| BP | GO:0034097 | Response to cytokine | 1.22E-12 |
| BP | GO:0009615 | Response to virus | 1.05E-11 |
| BP | GO:0009605 | Response to external stimulus | 1.10E-09 |
| BP | GO:0098542 | Defense response to other organism | 1.43E-09 |
| BP | GO:0051607 | Defense response to virus | 3.16E-09 |
| BP | GO:0051704 | Multi-organism process | 4.69E-09 |
| BP | GO:0060337 | Type i interferon signaling pathway | 5.01E-09 |
| BP | GO:0071357 | Cellular response to type i interferon | 5.01E-09 |
| BP | GO:0034340 | Response to type i interferon | 7.45E-09 |
| BP | GO:0002682 | Regulation of immune system process | 1.14E-08 |
| BP | GO:0050776 | Regulation of immune response | 4.36E-08 |
| BP | GO:0002252 | Immune effector process | 5.50E-08 |
| BP | GO:0007166 | Cell surface receptor signaling pathway | 4.80E-07 |
| BP | GO:0002684 | Positive regulation of immune system process | 1.35E-06 |
| BP | GO:0070098 | Chemokine-mediated signaling pathway | 7.87E-06 |
| BP | GO:1990868 | Response to chemokine | 1.56E-05 |
| BP | GO:1990869 | Cellular response to chemokine | 1.56E-05 |
| BP | GO:0007204 | Positive regulation of cytosolic calcium ion concentration | 2.56E-05 |
| BP | GO:0071310 | Cellular response to organic substance | 6.21E-05 |
| BP | GO:0051480 | Regulation of cytosolic calcium ion concentration | 7.18E-05 |
| BP | GO:0050896 | Response to stimulus | 8.36E-05 |
| BP | GO:0034341 | Response to interferon-gamma | 1.12E-04 |
| BP | GO:0001775 | Cell activation | 2.43E-04 |
| BP | GO:0006950 | Response to stress | 2.70E-04 |
| BP | GO:0045321 | Leukocyte activation | 3.13E-04 |
| BP | GO:0002250 | Adaptive immune response | 3.13E-04 |
| BP | GO:0045087 | Innate immune response | 4.64E-04 |
| BP | GO:0002520 | Immune system development | 4.93E-04 |
| BP | GO:0070887 | Cellular response to chemical stimulus | 5.19E-04 |
| BP | GO:0010033 | Response to organic substance | 5.54E-04 |
| BP | GO:0060402 | Calcium ion transport into cytosol | 5.67E-04 |
| BP | GO:0009617 | Response to bacterium | 6.33E-04 |
| BP | GO:0006874 | Cellular calcium ion homeostasis | 7.22E-04 |
| BP | GO:0001817 | Regulation of cytokine production | 7.56E-04 |
| BP | GO:0030593 | Neutrophil chemotaxis | 8.17E-04 |
| BP | GO:0001819 | Positive regulation of cytokine production | 8.30E-04 |
| BP | GO:0030098 | Lymphocyte differentiation | 9.06E-04 |
| BP | GO:0055074 | Calcium ion homeostasis | 9.33E-04 |
| BP | GO:0046649 | Lymphocyte activation | 1.03E-03 |
| BP | GO:0071346 | Cellular response to interferon-gamma | 1.03E-03 |
| BP | GO:0060401 | Cytosolic calcium ion transport | 1.15E-03 |
| BP | GO:0072503 | Cellular divalent inorganic cation homeostasis | 1.39E-03 |
| BP | GO:0050900 | Leukocyte migration | 1.49E-03 |
| BP | GO:1990266 | Neutrophil migration | 1.80E-03 |
| BP | GO:0048534 | Hematopoietic or lymphoid organ development | 1.92E-03 |
| BP | GO:0072507 | Divalent inorganic cation homeostasis | 1.99E-03 |
| BP | GO:0001816 | Cytokine production | 2.18E-03 |
| BP | GO:0071621 | Granulocyte chemotaxis | 2.18E-03 |
| BP | GO:0002521 | Leukocyte differentiation | 2.41E-03 |
| BP | GO:0051209 | Release of sequestered calcium ion into cytosol | 2.63E-03 |
| BP | GO:0051283 | Negative regulation of sequestering of calcium ion | 2.88E-03 |
| BP | GO:0051282 | Regulation of sequestering of calcium ion | 3.15E-03 |
| BP | GO:0051208 | Sequestering of calcium ion | 3.59E-03 |
| BP | GO:0030595 | Leukocyte chemotaxis | 4.67E-03 |
| BP | GO:0097530 | Granulocyte migration | 5.02E-03 |
| BP | GO:0097553 | Calcium ion transmembrane import into cytosol | 5.23E-03 |
| BP | GO:0032735 | Positive regulation of interleukin-12 production | 5.78E-03 |
| BP | GO:0006875 | Cellular metal ion homeostasis | 6.00E-03 |
| BP | GO:0050778 | Positive regulation of immune response | 6.95E-03 |
| BP | GO:0030097 | Hemopoiesis | 8.07E-03 |
| BP | GO:0060333 | Interferon-gamma-mediated signaling pathway | 8.07E-03 |
| BP | GO:0002819 | Regulation of adaptive immune response | 8.32E-03 |
| BP | GO:0030217 | T cell differentiation | 8.48E-03 |
| CC | GO:0009897 | External side of plasma membrane | 2.74E-09 |
| CC | GO:0098552 | Side of membrane | 2.96E-08 |
| CC | GO:0009986 | Cell surface | 7.21E-08 |
| CC | GO:0044459 | Plasma membrane part | 3.23E-03 |
| MF | GO:0048248 | CXCR3 chemokine receptor binding | 8.06E-08 |
| MF | GO:0045236 | CXCR chemokine receptor binding | 5.26E-06 |
| MF | GO:0042379 | Chemokine receptor binding | 6.91E-06 |
| MF | GO:0008009 | Chemokine activity | 6.59E-05 |
| MF | GO:0001664 | G protein-coupled receptor binding | 2.79E-03 |
| MF | GO:0005102 | Signaling receptor binding | 3.02E-03 |
| MF | GO:0005126 | Cytokine receptor binding | 3.28E-03 |
| MF | GO:0031735 | CCR10 chemokine receptor binding | 8.34E-03 |
| MF | GO:0005125 | Cytokine activity | 8.34E-03 |

**Supplementary Table 5. KEGG pathway enrichment analysis of common 51 DEGs.**

| **ID** | **GO Term** | ***adj.p*** | **Associated Genes** |
| --- | --- | --- | --- |
| KEGG:05340 | Primary immunodeficiency | 1.80E-06 | CD3D, ICOS, IL7R, PTPRC, TAP1 |
| KEGG:04062 | Chemokine signaling pathway | 2.90E-06 | CCL19, CCR7, CXCL10, CXCL11, CXCL13, CXCL9, CXCR4, STAT1 |
| KEGG:04640 | Hematopoietic cell lineage | 7.22E-06 | CD2, CD3D, CR2, HLA-DRA, IL7R, MS4A1 |
| KEGG:04514 | Cell adhesion molecules (CAMs) | 4.73E-05 | CD2, HLA-DRA, HLA-F, ICOS, PTPRC, SELL |
| KEGG:04672 | Intestinal immune network for IgA production | 1.07E-03 | CXCR4, HLA-DRA, ICOS |

­­

**Supplementary Table 6. The results of Single gene GSEA of High and Low expression of ICOS**

| **KEGG Pathway** | **ES** | **NES** | **NOM p-val** | **FDR q-val** |
| --- | --- | --- | --- | --- |
| Cytokine-cytokine receptor interaction | 0.581 | 1.936 | 0 | 3.98E-03 |
| Intestinal Immune Network For IgA Production | 0.775 | 1.930 | 0 | 4.05E-03 |
| Hematopoietic cell lineage | 0.706 | 1.922 | 0 | 4.13E-03 |
| Cell adhesion molecules (CAMs) | 0.566 | 1.897 | 0 | 4.90E-03 |
| Chemokine signaling pathway | 0.548 | 1.946 | 0 | 5.02E-03 |
| T cell receptor signaling pathway | 0.599 | 1.952 | 0 | 5.39E-03 |
| Natural killer cell mediated cytotoxicity | 0.609 | 1.957 | 0 | 6.19E-03 |
| Graft versus host disease | 0.870 | 1.863 | 0 | 6.20E-03 |
| Allograft rejection | 0.863 | 1.855 | 0 | 6.35E-03 |
| Nod like receptor signaling pathway | 0.567 | 1.851 | 6.19E-03 | 6.37E-03 |
| Cytosolic dna sensing pathway | 0.588 | 1.864 | 6.10E-03 | 6.64E-03 |
| B cell receptor signaling pathway | 0.597 | 1.796 | 6.05E-03 | 1.08E-02 |
| Fc epsilon ri signaling pathway | 0.514 | 1.798 | 3.98E-03 | 1.11E-02 |
| Antigen Processing and Presentation | 0.645 | 1.802 | 1.22E-02 | 1.12E-02 |
| Toll like receptor signaling pathway | 0.599 | 2.013 | 0 | 1.12E-02 |
| Primary immunodeficiency | 0.777 | 1.789 | 0 | 1.14E-02 |
| Jak stat signaling pathway | 0.486 | 1.757 | 0 | 1.56E-02 |
| Cell cycle | 0.433 | 1.716 | 2.10E-02 | 2.36E-02 |
| Apoptosis | 0.452 | 1.675 | 1.57E-02 | 3.53E-02 |
| Rig i like receptor signaling pathway | 0.461 | 1.656 | 1.39E-02 | 4.03E-02 |

Abbreviation: ES: enrichment score; NES: normalized enrichment score; FDR: false-discovery rate.

**Supplementary Table 7. Results of Immune infiltration analysis in salivary glands.**

| **Sample** | N1 | N2 | N3 | N4 | N5 | N6 | N7 | N8 | N9 | N10 | N11 | N12 | N13 | Y1 | Y2 | Y3 | Y4 | Y5 | Y6 | Y7 | Y8 | Y9 | Y10 | Y11 | Y12 | Y13 | Y14 | Y15 | Y16 |
| --- | --- | --- | --- | --- | --- | --- | --- | --- | --- | --- | --- | --- | --- | --- | --- | --- | --- | --- | --- | --- | --- | --- | --- | --- | --- | --- | --- | --- | --- |
| **B cell** | 0.043 | 0.134 | 0.045 | 0.064 | 0.071 | 0.098 | 0.06 | 0.137 | 0.019 | 0.2 | 0 | 0.056 | 0.037 | 0.062 | 0.351 | 0.186 | 0.211 | 0.076 | 0.071 | 0.081 | 0.069 | 0.206 | 0.157 | 0.38 | 0.165 | 0.152 | 0.03 | 0.127 | 0.101 |
| **CD4 T** | 0.038 | 0.049 | 0 | 0.031 | 0.134 | 0.005 | 0.031 | 0.232 | 0.004 | 0.165 | 0.037 | 0.057 | 0.015 | 0.024 | 0.135 | 0.127 | 0.162 | 0.031 | 0.026 | 0.049 | 0.016 | 0.131 | 0.06 | 0.151 | 0.164 | 0.126 | 0.075 | 0.043 | 0.035 |
| **CD4 naive** | 0.216 | 0.194 | 0.177 | 0.255 | 0.211 | 0.222 | 0.173 | 0.223 | 0.222 | 0.257 | 0.226 | 0.205 | 0.232 | 0.198 | 0.124 | 0.152 | 0.104 | 0.178 | 0.177 | 0.153 | 0.197 | 0.167 | 0.148 | 0 | 0.102 | 0.125 | 0.189 | 0.238 | 0.161 |
| **CD8 T** | 0.103 | 0.08 | 0.04 | 0.031 | 0.094 | 0.066 | 0.104 | 0.065 | 0.054 | 0.034 | 0.126 | 0.087 | 0.011 | 0.036 | 0.11 | 0.143 | 0.127 | 0 | 0.087 | 0.109 | 0.059 | 0.151 | 0.134 | 0.111 | 0.165 | 0.14 | 0.128 | 0.121 | 0.103 |
| **CD8 naive** | 0.007 | 0.017 | 0.016 | 0 | 0 | 0.036 | 0.038 | 0 | 0.041 | 0.071 | 0.068 | 0.037 | 0 | 0.011 | 0 | 0 | 0 | 0.01 | 0.014 | 0.03 | 0.031 | 0.029 | 0.001 | 0 | 0 | 0 | 0.05 | 0.022 | 0.018 |
| **Central memory** | 0 | 0 | 0.006 | 0.061 | 0 | 0.039 | 0 | 0 | 0.006 | 0 | 0 | 0.024 | 0.012 | 0.009 | 0.104 | 0.087 | 0.15 | 0.015 | 0.034 | 0.048 | 0.008 | 0.042 | 0.027 | 0.005 | 0 | 0 | 0.061 | 0.022 | 0 |
| **Cytotoxic** | 0.049 | 0.072 | 0 | 0 | 0.16 | 0.033 | 0.135 | 0.103 | 0.019 | 0.041 | 0.021 | 0.108 | 0 | 0 | 0.044 | 0.077 | 0.108 | 0 | 0.064 | 0.113 | 0.04 | 0.084 | 0.071 | 0.207 | 0.193 | 0.186 | 0.035 | 0.113 | 0.073 |
| **DC** | 0.164 | 0.173 | 0.197 | 0.124 | 0.141 | 0.178 | 0.049 | 0.165 | 0.158 | 0.032 | 0.101 | 0 | 0.234 | 0.106 | 0.207 | 0.162 | 0.284 | 0.288 | 0.179 | 0.187 | 0.201 | 0.161 | 0.148 | 0.329 | 0.347 | 0.251 | 0.144 | 0.202 | 0.229 |
| **Effector_memory** | 0.135 | 0.138 | 0.144 | 0.113 | 0.159 | 0.143 | 0.148 | 0 | 0.129 | 0 | 0.154 | 0.092 | 0.105 | 0.146 | 0.187 | 0.161 | 0.139 | 0.159 | 0.142 | 0.163 | 0.147 | 0.128 | 0.176 | 0.136 | 0.178 | 0.132 | 0.114 | 0.128 | 0.187 |
| **Exhausted** | 0 | 0 | 0 | 0 | 0 | 0 | 0 | 0 | 0 | 0 | 0 | 0 | 0 | 0 | 0.025 | 0.02 | 0.002 | 0 | 0 | 0 | 0 | 0.004 | 0 | 0.104 | 0.063 | 0.039 | 0 | 0 | 0 |
| **Gamma_delta** | 0.077 | 0.088 | 0.061 | 0.078 | 0.186 | 0.043 | 0.132 | 0.157 | 0.065 | 0.132 | 0.137 | 0.088 | 0.006 | 0.042 | 0.01 | 0.03 | 0.04 | 0 | 0.068 | 0.081 | 0.105 | 0.062 | 0.082 | 0.203 | 0.222 | 0.191 | 0.126 | 0.071 | 0.092 |
| **InfiltrationScore** | 0.624 | 0.656 | 0.542 | 0.485 | 0.692 | 0.494 | 0.401 | 0.892 | 0.492 | 0.803 | 0.458 | 0.424 | 0.556 | 0.457 | 0.616 | 0.631 | 0.727 | 0.605 | 0.553 | 0.585 | 0.569 | 0.741 | 0.567 | 0.831 | 0.9 | 0.785 | 0.558 | 0.611 | 0.65 |
| **MAIT** | 0.063 | 0.051 | 0.006 | 0 | 0.048 | 0 | 0.001 | 0 | 0.026 | 0 | 0.003 | 0.061 | 0.008 | 0 | 0.09 | 0.117 | 0.064 | 0 | 0.063 | 0.052 | 0.062 | 0.103 | 0.113 | 0.051 | 0.101 | 0.101 | 0.052 | 0.078 | 0.078 |
| **Macrophage** | 0.195 | 0.193 | 0.169 | 0.103 | 0.182 | 0.037 | 0.067 | 0.167 | 0.108 | 0.18 | 0.058 | 0 | 0.115 | 0.116 | 0.071 | 0.146 | 0.103 | 0.246 | 0.108 | 0.121 | 0.178 | 0.155 | 0.13 | 0.288 | 0.364 | 0.294 | 0.071 | 0.158 | 0.243 |
| **Monocyte** | 0.238 | 0.269 | 0.204 | 0.205 | 0.238 | 0.19 | 0.177 | 0.26 | 0.215 | 0.247 | 0.207 | 0.222 | 0.254 | 0.213 | 0.087 | 0.169 | 0.173 | 0.205 | 0.225 | 0.224 | 0.214 | 0.159 | 0.163 | 0 | 0.124 | 0.128 | 0.243 | 0.168 | 0.182 |
| **NK** | 0.088 | 0.018 | 0.071 | 0.038 | 0.116 | 0.045 | 0.005 | 0.211 | 0.059 | 0.216 | 0.036 | 0.074 | 0.067 | 0.005 | 0.031 | 0.005 | 0.056 | 0.004 | 0.05 | 0.066 | 0 | 0.103 | 0.054 | 0.07 | 0.096 | 0.094 | 0.063 | 0.034 | 0.064 |
| **NKT** | 0.146 | 0.029 | 0.097 | 0.079 | 0.155 | 0.016 | 0.113 | 0.204 | 0.117 | 0.259 | 0.13 | 0.102 | 0.139 | 0.063 | 0 | 0 | 0.033 | 0.005 | 0.073 | 0.008 | 0.085 | 0.068 | 0 | 0 | 0 | 0.105 | 0.057 | 0.088 | 0.016 |
| **Neutrophil** | 0.137 | 0.141 | 0.148 | 0.185 | 0.138 | 0.177 | 0.153 | 0.2 | 0.175 | 0.22 | 0.174 | 0.188 | 0.163 | 0.174 | 0 | 0.078 | 0.055 | 0.125 | 0.145 | 0.106 | 0.18 | 0.128 | 0.07 | 0.009 | 0.026 | 0.08 | 0.145 | 0.132 | 0.09 |
| **Tfh** | 0.003614 | 0.029 | 0 | 0 | 0.001059 | 0.025 | 0 | 0.019646 | 0 | 0 | 0 | 0.041 | 0.024 | 0 | 0.241 | 0.198 | 0.243 | 0 | 0 | 0.096 | 0.006 | 0.164 | 0.158 | 0.268 | 0.278 | 0.192 | 0.161 | 0.058 | 0.056 |
| **Th1** | 0.142 | 0.108 | 0.163 | 0.171 | 0.112 | 0.164 | 0.123 | 0 | 0.163 | 0 | 0.087 | 0.172 | 0.171 | 0.229 | 0.183 | 0.236 | 0.164 | 0.194 | 0.182 | 0.191 | 0.192 | 0.125 | 0.132 | 0.207 | 0.132 | 0.12 | 0.184 | 0.13 | 0.192 |
| **Th17** | 0 | 0 | 0.002402 | 0.013257 | 0 | 0.024354 | 0.014 | 0 | 0.03 | 0 | 0.028 | 0.012222 | 0.027 | 0.042 | 0.044 | 0.032 | 0.059 | 0.037 | 0.031 | 0.026 | 0 | 0 | 0.023 | 0.029525 | 0.033746 | 0.029664 | 0.052 | 0.014993 | 0.024787 |
| **Th2** | 0.138 | 0.112 | 0.182 | 0.063 | 0.151 | 0.043 | 0.073 | 0 | 0.098 | 0 | 0.106 | 0.061 | 0.052 | 0.076 | 0.122 | 0.067 | 0.066 | 0.148 | 0.205 | 0.174 | 0.108 | 0.1 | 0.201 | 0.009 | 0.041 | 0.073 | 0.148 | 0.14 | 0.123 |
| **Tr1** | 0.043 | 0.243 | 0 | 0 | 0.195 | 0.026 | 0.094 | 0.172 | 0 | 0.078 | 0 | 0.058 | 0 | 0 | 0.16 | 0.193 | 0.254 | 0.087 | 0.053 | 0.06 | 0 | 0.08 | 0.133 | 0.306 | 0.273 | 0.257 | 0.017 | 0.13 | 0.139 |
| **iTreg** | 0.06 | 0 | 0 | 0.029 | 0.107 | 0 | 0.016 | 0.115 | 0 | 0.124 | 0 | 0.103 | 0.059 | 0.027 | 0.038 | 0.037 | 0.139 | 0 | 0 | 0.016 | 0 | 0 | 0.029 | 0 | 0.044 | 0.07 | 0.015 | 0 | 0 |
| **nTreg** | 0 | 0 | 0 | 0.042 | 0.033 | 0 | 0 | 0.025 | 0 | 0.03 | 0.033 | 0.035 | 0 | 0 | 0 | 0 | 0 | 0 | 0 | 0 | 0 | 0 | 0 | 0 | 0.016 | 0 | 0 | 0.004 | 0 |

**Supplementary Figure1. Quality control of gene expression, principal component analysis (PCA) and heatmap of 51 common DEGs.**

**Supplementary Figure2. Identification of modules and hub genes closely associated with clinical traits of pSS in RNA-seq data through WGCNA. A.** The left figure indicates the scale-free fit index (R^2) and the right panel shows the mean connectivity for various soft-thresholding powers. **B.** The clustering dendrograms of module eigengenes and the red line represents cut height (0.25); **C.** The clustering heatmap of the correlation among Focus score ≥ 1and different modules. **D.** Heatmap of randomly selected 1000 genes showing correlation among each module.

**Supplementary Figure3. Circular visualization of chromosomal locations and expression of 51 common DEGs.** The outer circle represents chromosomes and lines attached to the genes indicate their specific chromosomal locations. The inner circle represents gene expression of DEGs in microarray data of pSS+non-pSS vs Control (A), microarray data of pSS vs non-pSS (B) and RNA-seq data of pSS vs non-pSS (C). The seven hub genes were shown in red and connected with red lines in the circle’s center.

**Supplementary Figure4. The expression levels of *ICOS* in various tissues from the healthy based on GTEx database.** The figure was drawn employing the website: <http://www.gtexportal.org>.

**Supplementary Figure5. The results of *ICOS* expression based on different clinical phenotypes and ELISA for inflammatory factor without significant difference. A-G.** The expression levels of *ICOS* in different subgroups in SGs RNA-seq (16 pSS and 13 non-pSS) based on Xerostomia positive, Focus score ≥1, anti-SSA/Ro60 positive, High IgG, High ESR, Ro52 positive and Xerophthalmia; **H-K.** The expression levels of *ICOS* in different subgroups in SGs RT-qPCR (58 pSS) based on anti-SSA positive, High ESR, Low C3 and KCS positive. **L-N.** The concentration levels of TGFβ1, IL-6 and IL-4 in plasma of pSS through ELISA.
