# Supplementary figures and images for: Transcriptome Sequencing Reveals Potential Roles of ICOS in Primary Sjögren’s Syndrome
Source: Front Cell Dev Biol. 2020 Dec 4;8:592490. doi: 10.3389/fcell.2020.592490 (PMC7747463; doi:10.3389/fcell.2020.592490)

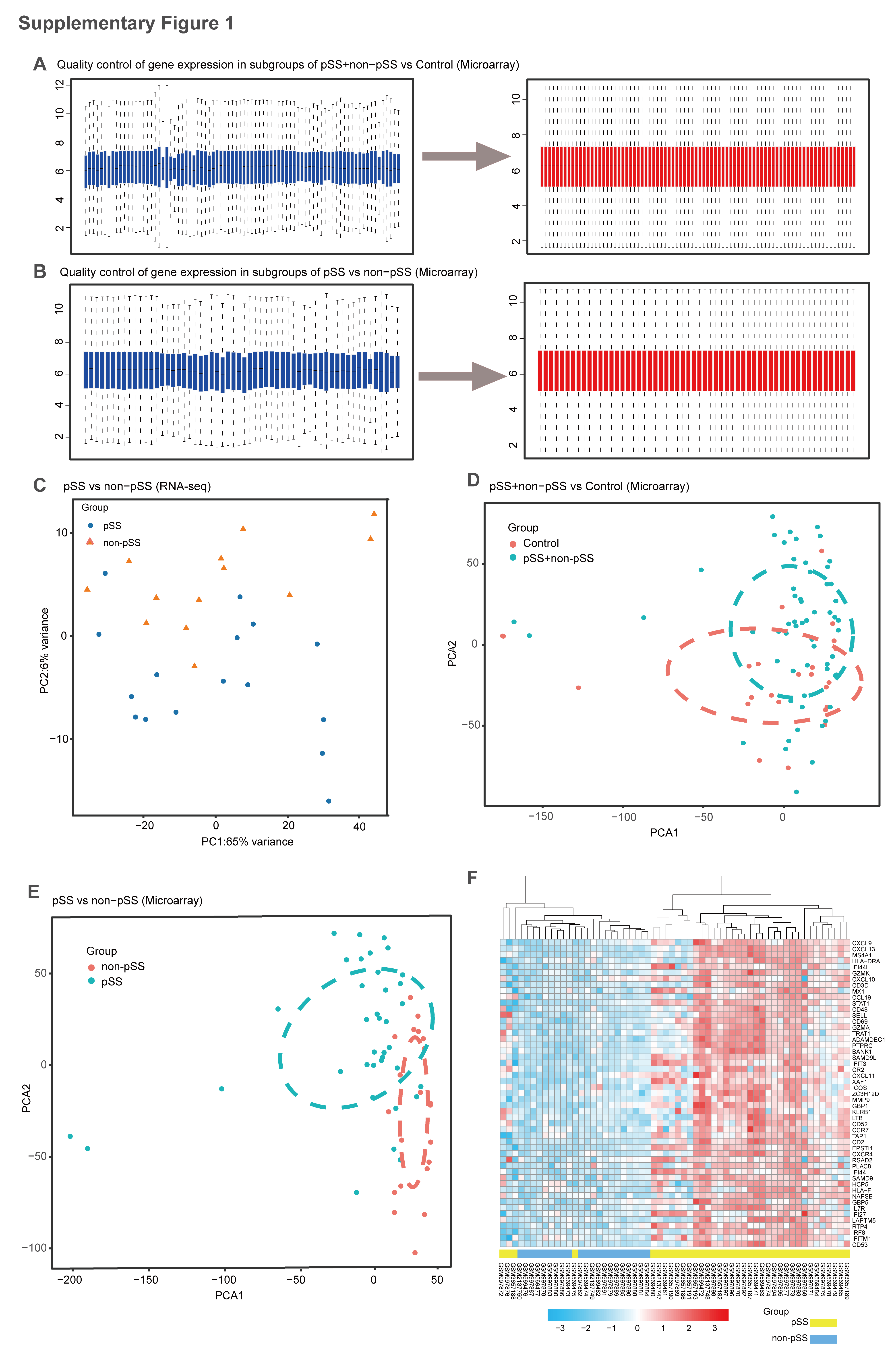

Supplement: Supplementary file 1 [file Data_Sheet_1.zip › Supplementary Material/Supplementary Figure 1.tif]

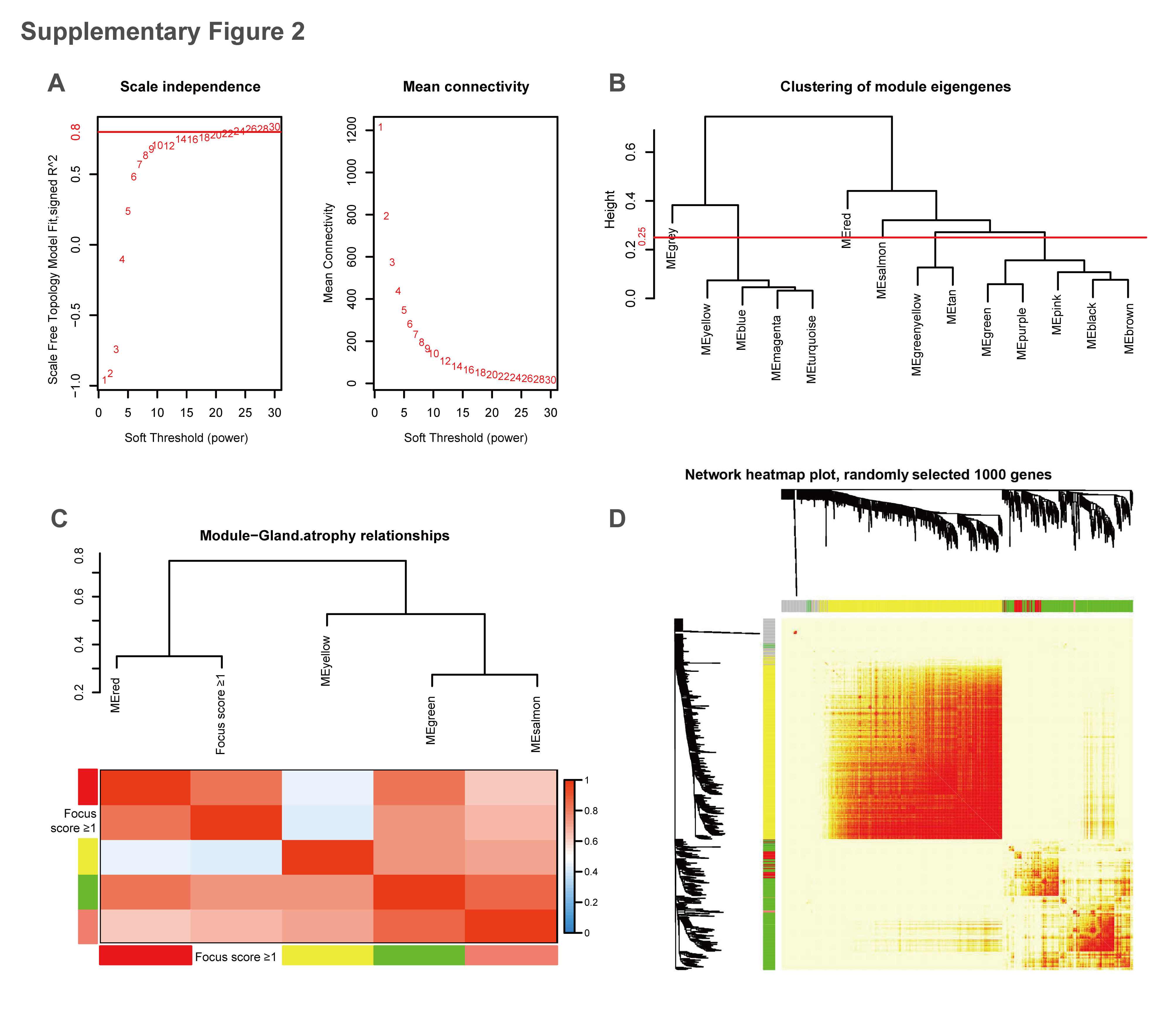

Supplement: Supplementary file 1 [file Data_Sheet_1.zip › Supplementary Material/Supplementary Figure 2.tif]

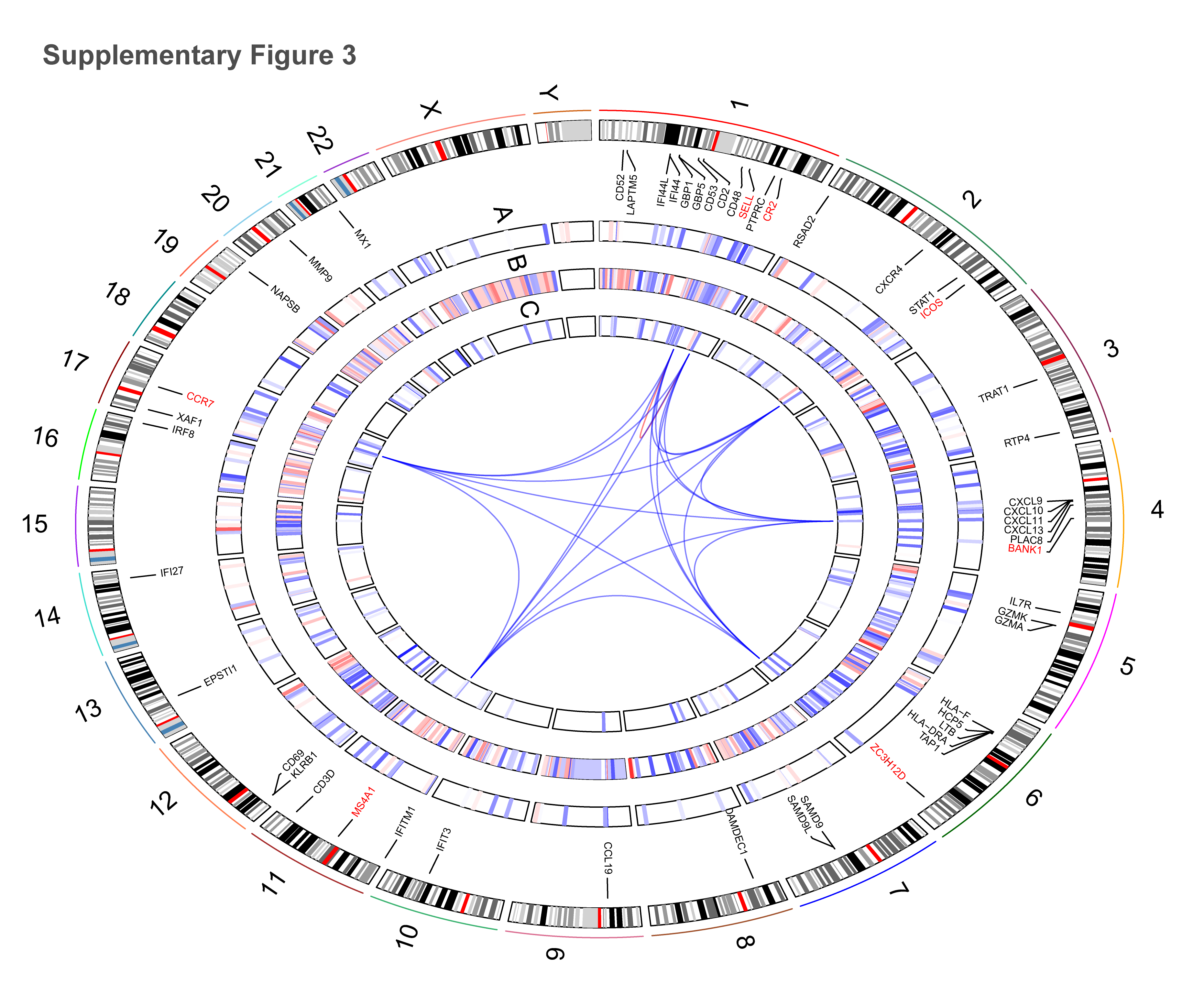

Supplement: Supplementary file 1 [file Data_Sheet_1.zip › Supplementary Material/Supplementary Figure 3.tif]

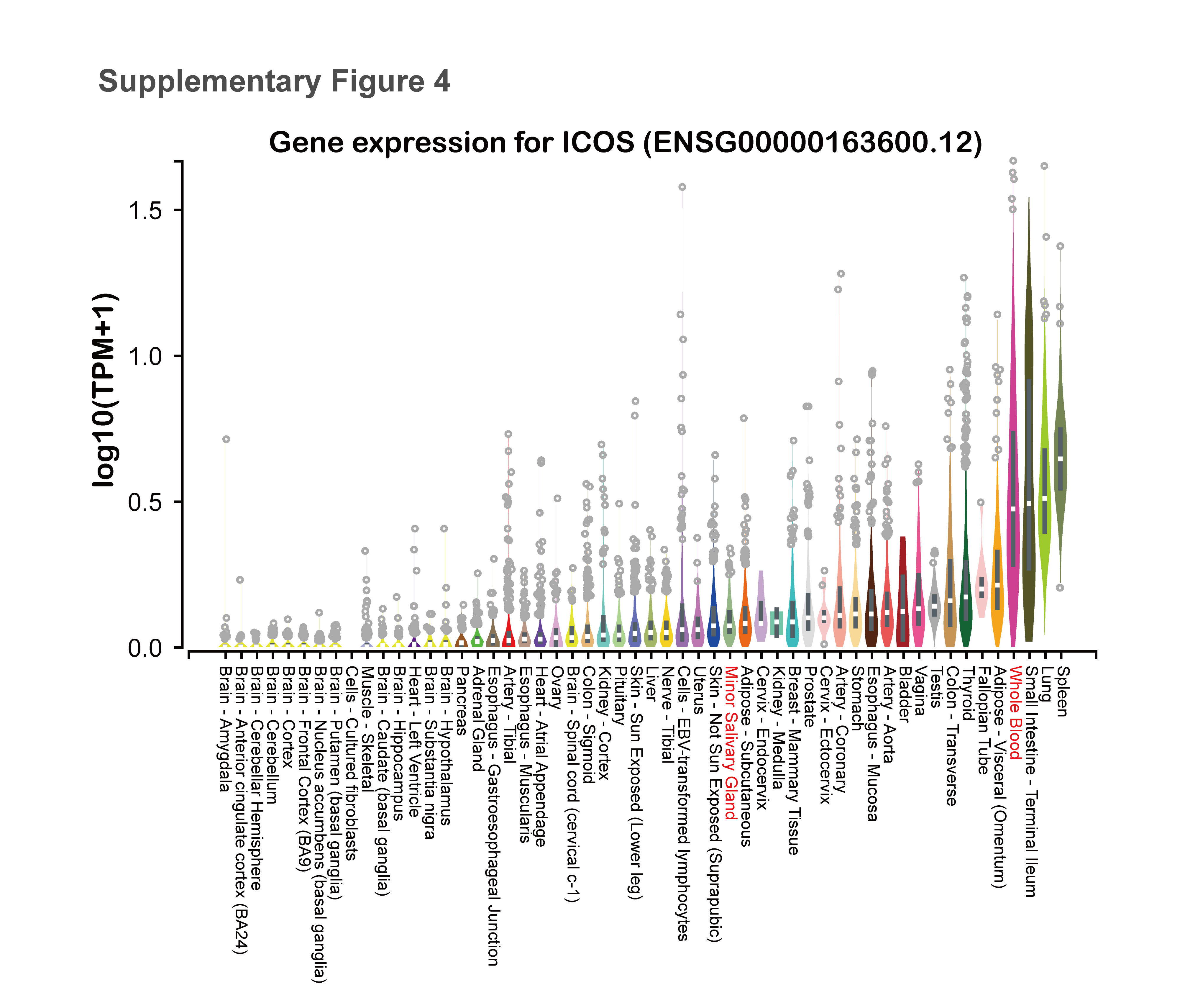

Supplement: Supplementary file 1 [file Data_Sheet_1.zip › Supplementary Material/Supplementary Figure 4.tif]

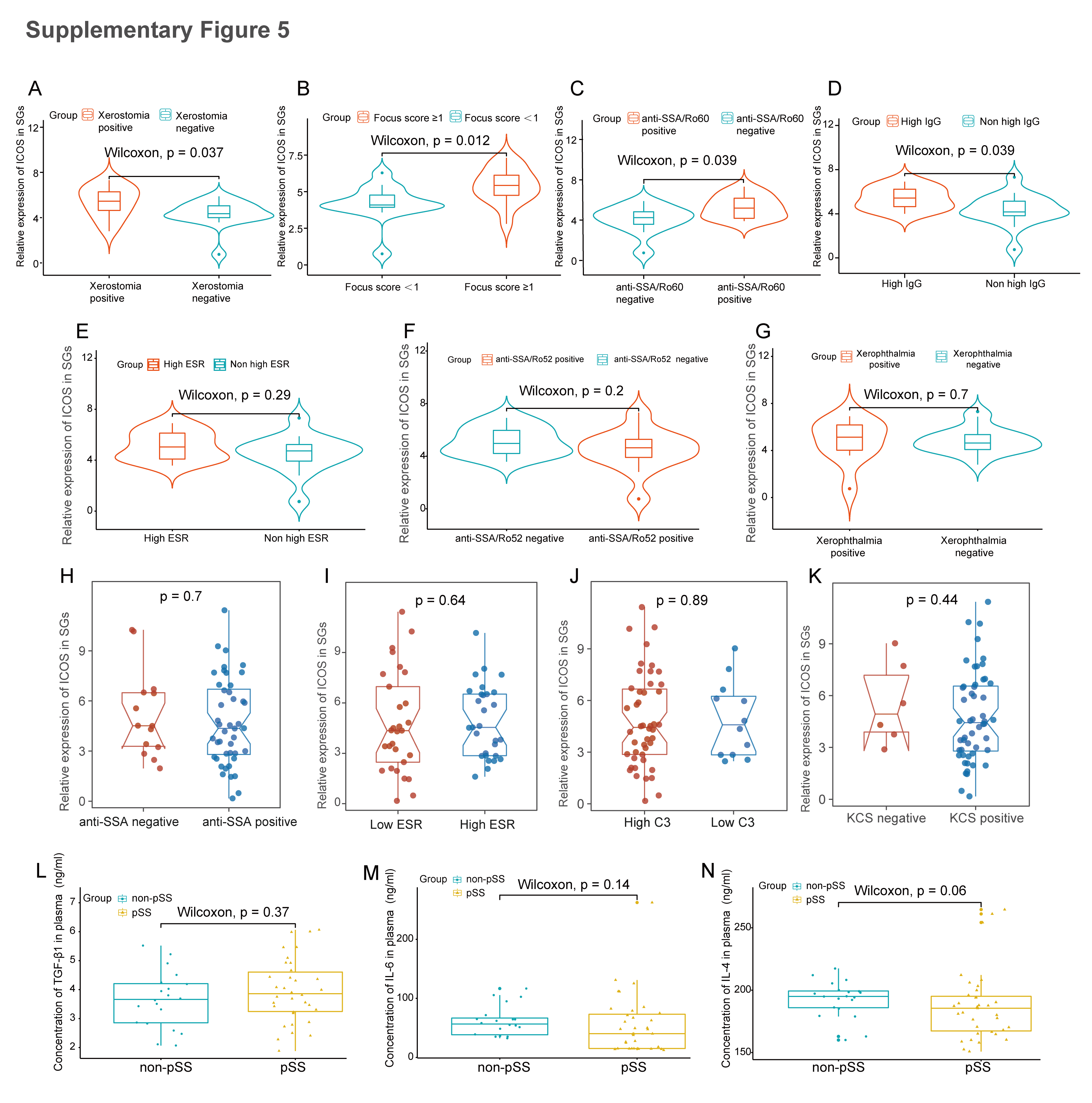

Supplement: Supplementary file 1 [file Data_Sheet_1.zip › Supplementary Material/Supplementary Figure 5.tif]
